# Scalability of nanopore osmotic energy conversion

**DOI:** 10.1002/EXP.20220110

**Published:** 2024-01-08

**Authors:** Makusu Tsutsui, Wei‐Lun Hsu, Kazumichi Yokota, Iat Wai Leong, Hirofumi Daiguji, Tomoji Kawai

**Affiliations:** ^1^ The Institute of Scientific and Industrial Research Osaka University Ibaraki Osaka Japan; ^2^ Department of Mechanical Engineering The University of Tokyo Bunkyo‐ku Tokyo Japan; ^3^ Health and Medical Research Institute National Institute of Advanced Industrial Science and Technology (AIST) Takamatsu Kagawa Japan

**Keywords:** interpore interactions, ion selectivity, multipore, osmotic power, reverse electrodialysis

## Abstract

Artificial nanofluidic networks are emerging systems for blue energy conversion that leverages surface charge‐derived permselectivity to induce voltage from diffusive ion transport under salinity difference. Here the pivotal significance of electrostatic inter‐channel couplings in multi‐nanopore membranes, which impose constraints on porosity and subsequently influence the generation of large osmotic power outputs, is illustrated. Constructive interference is observed between two 20 nm nanopores of 30 nm spacing that renders enhanced permselectivity to osmotic power output via the recovered electroneutrality. On contrary, the interference is revealed as destructive in two‐dimensional arrays causing significant deteriorations of the ion selectivity even for the nanopores sparsely distributed at an order of magnitude larger spacing than the Dukhin length. Most importantly, a scaling law is provided for deducing the maximal membrane area and porosity to avoid the selectivity loss via the inter‐pore electrostatic coupling. As the electric crosstalk is inevitable in any fluidic network, the present findings can be a useful guide to design nanoporous membranes for scalable osmotic power generations.

## Graphical abstract

Achieving scalability remains a pivotal hurdle in the realm of osmotic power generation utilizing nanoporous membranes. This study addresses this challenge by introducing a scaling law that facilitates the estimation of the maximum membrane area and porosity achievable without compromising permselectivity due to inter‐pore electrostatic couplings.

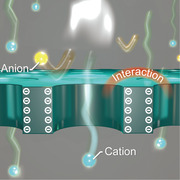

## 1 INTRODUCTION

Salt concentration difference induces a unidirectional flow of ions through conduits in a membrane. It establishes a mechanism for converting Gibbs free energy into electricity by exploiting permselectivity of nanofluidic channels originating from the capability of surface charges at the walls to block the entrance of coions via electrostatic repulsions.^[^ ^1^, ^2^, ^3^, ^4^ ^]^ This osmotic power generation represents a promising avenue for harnessing “blue energy” from sea and freshwater with potential energy yields exceeding 2 MJ m^−3^.^[^ ^5^, ^6^ ^]^ Miscellaneous compounds of various structures have been examined to gain higher osmotic energy conversion efficiencies.^[^ ^7^, ^8^ ^]^ Of central challenge has been the quest to identify a membrane with high ion permeability and selectivity.^[^ ^9^ ^]^ In this context, atomic sheet motifs of two‐dimensional materials were promising for attaining large ionic conductance,^[^ ^10^, ^11^, ^12^ ^]^ which was actually demonstrated to potentially yield giant osmotic power at MW m^−2^ level under an envisaged porosity with the assumed linear scaling of the single‐pore performance.^[^ ^13^ ^]^ In contrast to the nanoelectromechanical system (NEMS) approach to sculpt a single nanopore, self‐organized nanoporous membranes offer chemically‐defined networks of nanofluidic channels at a bulk scale, and hence more compatible with real applications.^[^ ^14^, ^15^, ^16^, ^17^ ^]^ In this field, significant refinement of the fluidic designs has led to remarkable enhancements of osmotic power densities surpassing 100 W m^−2^.^[^ ^17^ ^]^ Yet, there is still a huge gap between the performances of a single‐nanopore and a porous membrane highlighting a crucial factor that considerably degrades the ability of the nanofluidic osmotic power generators when put in a form of multipore.^[^ ^18^, ^19^, ^20^ ^]^

**FIGURE 1:**
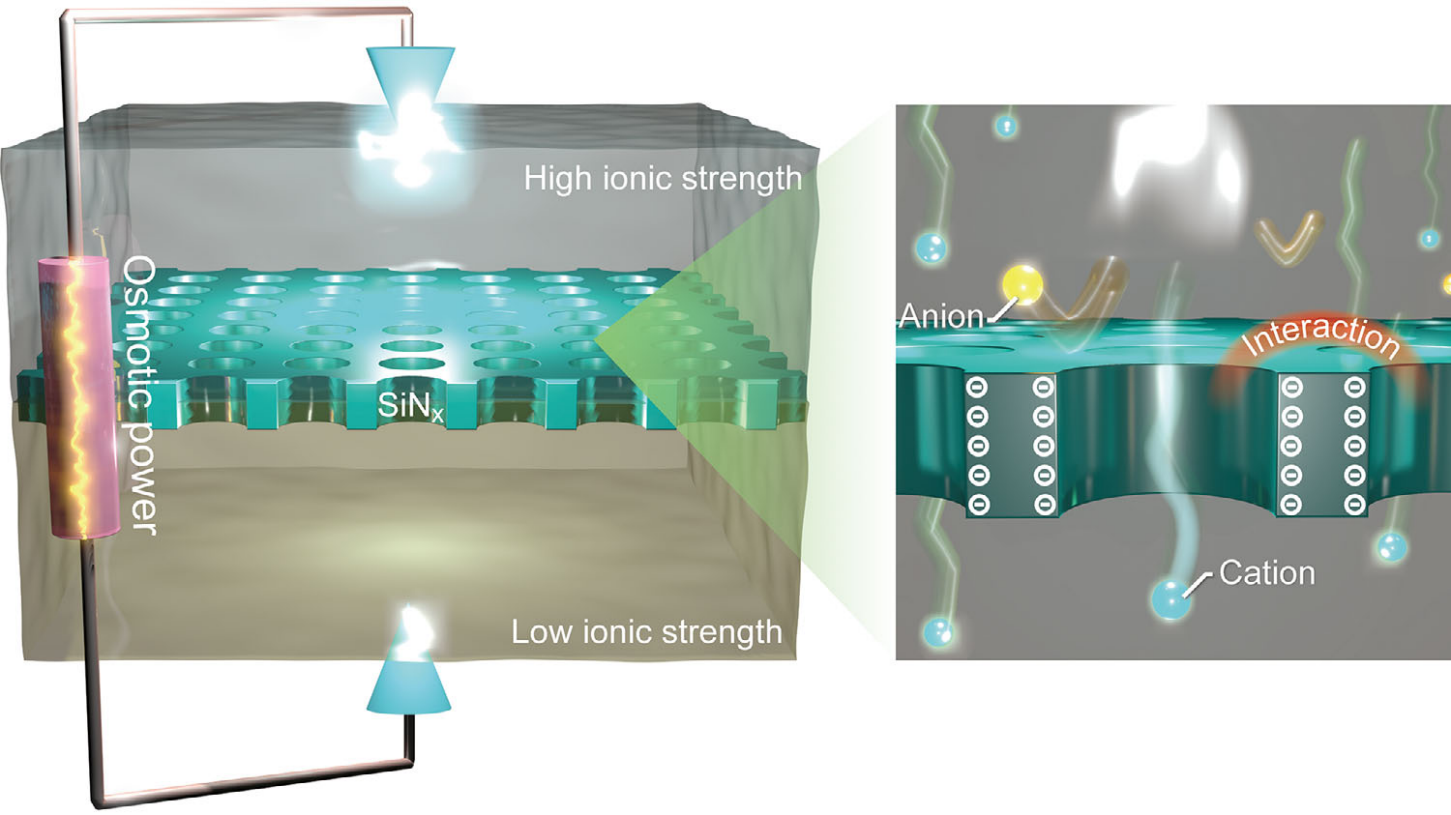
Nanofluidic osmotic power generation. Schematic model of multi‐nanopore (left) in an ultrathin SiNx membrane and a magnified view (right) depicting the charge‐selective ion transport via diffusion. Pore‐pore interactions are considered to significantly impair the energy conversion efficiency.

Recent efforts have thus been directed at exploring scalable nanofluidic network designs toward the practical applications,^[^ ^18^ ^]^ where smaller channels were reported as promising for attaining larger osmotic power density with the ability to maintain their high ion selectivity in hypersaline environments.^[^ ^21^ ^]^ Meanwhile, what remains to be elucidated is the role of inter‐channel coupling within nanofluidic networks particularly those formed in ultrathin membranes.^[^ ^1^, ^10^, ^22^, ^23^, ^24^ ^]^ This crucial issue becomes apparent as it encompasses various phenomena with distinct effective lengths. For example, short‐range electrostatic interactions in nanoscale proximity of closely‐packed nanochannels were reported to enhance the ion selectivity and conductivity^[^ ^17^ ^]^ owing to the restored electroneutrality.^[^ ^25^ ^]^ Conversely, recent experiments suggested long‐range interference between nanopores separated by sub‐micrometer distance inferring interplay between surface conductance and ion concentration polarization resulting in a rapid decline in the osmotic power.^[^ ^26^, ^27^ ^]^ To unravel the intriguing roles of the ionic crosstalk, we systematically evaluated the spacing‐dependent relationship between ion selectivity and osmotic power outputs in multi‐nanopore structures of varying arrangements each defined using electron beam lithographically (Figure 1).

## 2 RESULTS AND DISCUSSION

### 2.1 Permselectivity of single SiNx nanopores

**FIGURE 2:**
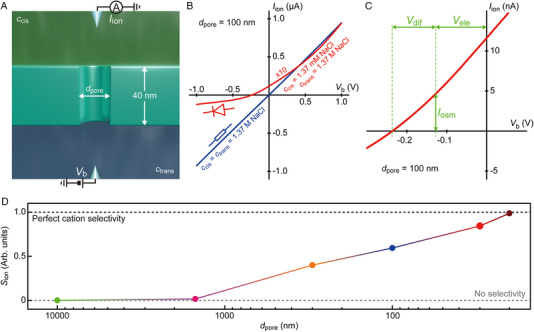
Channel size dependence of the permselectivity in single pores under salt gradients. (A) Schematic model depicting a cylindrical pore of diameter *d*_pore_ in a 40 nm‐thick SiN*_x_* membrane. The *cis* and the *trans* chambers are filled with phosphate buffered saline of NaCl concentrations *c_cis_* and *c_trans_*, respectively. (B) Ionic current *I*_ion_ through a 100 nm‐sized nanopore plotted as a function of the transmembrane voltage *V*_b_. The curve is straight and symmetric when there is no salt difference across the membrane (blue). In contrast, the characteristics becomes nonlinear and asymmetric with salt gradients (red; the current is shown as 10*I*_ion_). (C) A magnified view of (B) around zero voltage. The salinity difference tends to shift the ionic current curves due to the induced electrode potential difference (*V*_ele_) and diffusion potential (*V*_dif_). *I*_osm_ is the current induced by the diffusion potential‐derived ion transport. (D) The selectivity factor *S*_ion_ of single pores. Grey and black dashed lines point to *S*_ion_ = 0 and 1 denoting non‐ and perfect anion selectivity in the fluidic channels, respectively.

We start with a single cylindrical channel in a 40 nm‐thick SiN*_x_* membrane (Figure 2A). The pore diameter *d*_pore_ was varied in a range from 10 µm to 20 nm. Measuring the ionic current *I*_ion_ under the voltage sweep *V*_b_, we observed ohmic behavior irrespective of the pore size when *cis* and *trans* were filled with phosphate‐buffered saline (pH 7.4) of 1.37 m NaCl (Figure 2B, see also Figures S1–S7). This indicated the field‐driven flow of ions with constant mobilities and concentration in water as described by Maxwell's model.^[^ ^28^, ^29^ ^]^ However, when salinity gradients were introduced, the characteristics deviated from linearity, manifesting as rectifications in the ionic current. In the meantime, we detected positive offsets at *V*_b_ = 0 originating from the ion diffusion‐mediated voltage *V*_dif_ across the permselective nanochannel coupled with the redox potential difference *V*_ele_ at the electrodes (Figure 2C, see also Table S1).^[^ ^30^ ^]^

The asymmetric *I*_ion_—*V*_b_ characteristics offer a rich source of insights into the ion transport mechanism. The isoelectric point of SiN*_x_* suggests the presence of negative surface charges on the membrane in the salt solution.^[^ ^31^ ^]^ Anions are thus anticipated to be repelled and blocked at the orifice via the electrostatic repulsion rendering cation selectivity to the pores. The positive short circuit current represents this fact as a result of the selective ion flow via diffusion under the salinity gradients.^[^ ^11^ ^]^ It also predicts *I*_ion_ suppression at negative *V*_b_ for the electrostatic gating obstructs the transport of major carriers, that is, the high‐concentration cations at *trans*. Meanwhile, since the effects of ionic screening vary by the ion concentration conditions, the fluidic channels exhibited various ionic current characteristics (Figure S8) depending on *d*_pore_ as well as the ionic strength at *cis* (*c_cis_*) and *trans* (*c_trans_*).

In case of the 10 µm diameter pore, for instance, the rectification ratio *r*_rec_ at ±0.8 V remained close to 1 across the entire range of *c_cis_* from 1.37 m to 1.37 mm with *c_trans_* held constant at 1.37 m manifesting little influence of the surface charges for the size of the channel is more than three orders of magnitude larger than the Debye screening length *λ*_Debye_ (≤8 nm at *c_cis_* ≥ 1.37 mm). In contrast, the 1.5 µm channel showed diode‐like behaviors despite that *d*_pore_ >> *λ*_Debye_. We note the profound contribution of surface conductance to induce permselectivity even in conduits with non‐overlapped electric double layers, whose effectiveness is described by the Dukhin length *L*_D_ = *σ*/2*ec_cis_*, where *σ* and *e* are, respectively, the membrane surface charge density and the elementary charge.^[^ ^11^, ^32^ ^]^ Nevertheless, the micropore is still larger than *L*_D_ (= 57 nm at *c_cis_* = 1.37 mm with −15 × 10^−3^ C m^−2^ surface charge density for SiN*_x_*) leaving little room to consider the selective ion transport mechanism. This is also shown more directly by the permselectivity estimated from the salinity gradient‐dependent ionic current characteristics (Figure 2D). Theoretically, the diffusion voltage is given as *V*_dif_ = *S*_ion_(*k*_B_ *T*/*e*)ln(*a_cis_*/*a_trans_*),^[^ ^1^ ^]^ where *S*_ion_ is the selectivity factor denoting perfect anion (cation) selectivity when *S*_ion_ = +1 (−1) or non‐selective transport with *S* = 0. Here, *V*_dif_ was obtained by subtracting *V*_ele_ from the open circuit voltage, which allowed the estimations of *S*_ion_ under an approximation of *a_trans_*/*a_cis_* = *c_trans_*/*c_cis_*,^[^ ^13^ ^]^ where *a_cis_* and *a_trans_* are the ion activities at the *cis* and *trans* electrodes, respectively (Figure S9). As expected, *S*_ion_ is close to zero at *d*_pore_ = 1.5 µm signifying the non‐selective nature of the ion transport in the micropore.

Besides the permselectivity, electroosmosis can give ionic rectifications by pumping the low‐ (high‐) ion concentration solution of high (low) resistivity into the microchannel via the water flow directing from *cis* to *trans* (*trans* to *cis*) under negative (positive) transmembrane voltage.^[^ ^33^ ^]^ To visualize this phenomenon, the spatial ion concentration distributions were estimated by numerically solving the coupled Poisson‐Nernst‐Planck and Navier–Stokes equations in a framework of a finite element method (Figures S10 and S11).^[^ ^34^ ^]^ The *V*_b_‐dependent hydrodynamic flow was found to deplete (enrich) the ion concentration around the orifices, thereby inducing the diode‐like characteristics via the enlarged (diminished) Hall's access resistance^[^ ^35^ ^]^ (note the low depth‐to‐diameter aspect ratio structure of the micropore that makes the resistance outside the pore to be predominant^[^ ^27^, ^28^ ^]^).

As the pores were made smaller, on the other hand, this electroosmosis‐mediated ionic current rectification became weaker. At the same time, *r*_rec_ at large salt concentration ratio *c_trans_*/*c_cis_* tended to be higher reaching over 100 for *d*_pore_ = 20 nm. Under such low‐*c_cis_* conditions, the electrostatic repulsion from the negatively‐charged SiN*_x_* effectively impedes the anion transport in smaller conduits. The associated ion‐selective transport, which is marked by the positive *S*_ion_ at *d*_pore_ < 300 nm, anticipates ionic rectifications considering the little contributions of the high‐concentration ions at the *trans* to *I*_ion_ under negative transmembrane voltages. The overall results demonstrate the distinct nanopore size dependence of the electroosmosis and permselectivity effects on the asymmetric ion transport characteristics.

### 2.2 Inter‐pore interactions in double‐nanopore systems

**FIGURE 3:**
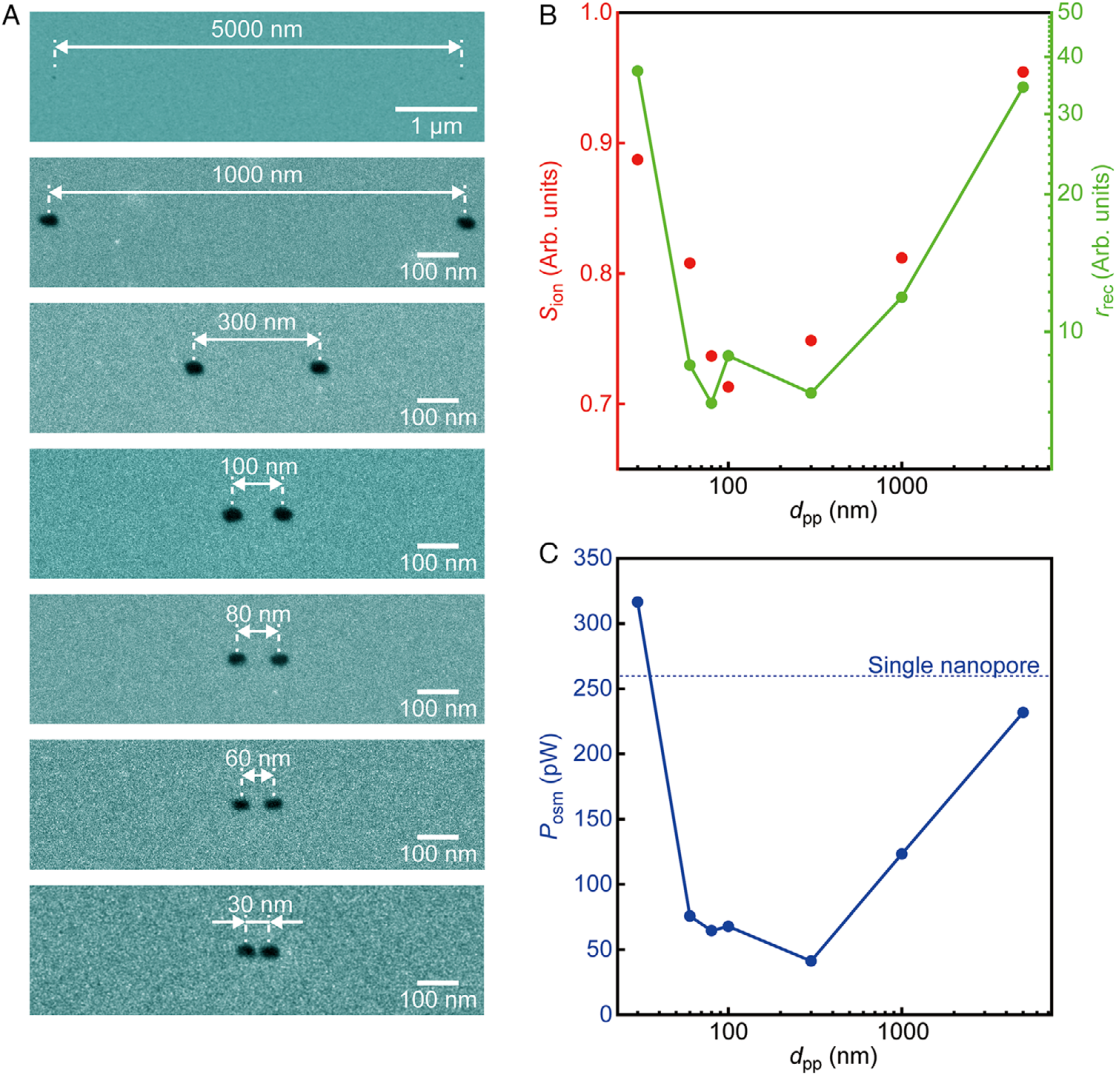
Inter‐pore interactions in pair‐pore systems. (A) False‐colored scanning electron micrographs of pair‐pore structures consisting of cylindrical channels of 20 nm diameter in a 40 nm‐thick SiN*_x_* membrane with the distance between the centers of the nanopores *d*_pp_ varied from 5000 to 30 nm. Note the lower magnification for the image of the nanopores separated by 5000 nm. (B) The inter‐pore distance dependence of *S*_ion_ (red) and *r*_rec_ under 1000‐fold salt gradients (green). (C) The osmotic power *P*_osm_ plotted against *d*_pp_. Dashed line is *P*_osm_ of two individual 20 nm‐sized nanopores.

The experiment was extended to examine the influence of inter‐channel interactions by investigating the salt gradient‐mediated ion transport through two 20 nm‐sized nanopores of various interpore distance *d*_pp_ (Figure 3A, see also Figures S12–S18). The salinity dependence of the rectifying behaviors in these pair‐pore structures were similar to those observed in the single nanopore (Figure S19) except for the quantitative difference at large *c_trans_*/*c_cis_*, where *r*_rec_ first decreased with diminishing the interpore distance from 5000 nm down to 300 nm, but then turned to increase by approaching the pores closer (green plots in Figure 3B). *S*_ion_ followed the non‐trivial dependence of *r*_rec_ indicating a profound effect of the pore‐pore interactions on the anion selectivity (red plots in Figure 3B; see also Figures S20–S22). Particularly, the enhanced selectivity at *d*_pp_ = 30 nm is promising in viewpoints of osmotic power generations (Figure 3C), which would be attributed to the recovered electroneutrality in the electrostatically‐coupled closely‐spaced nanopores.^[^ ^25^ ^]^

### 2.3 Osmotic energy conversion efficiency in multinanopore membranes

**FIGURE 4:**
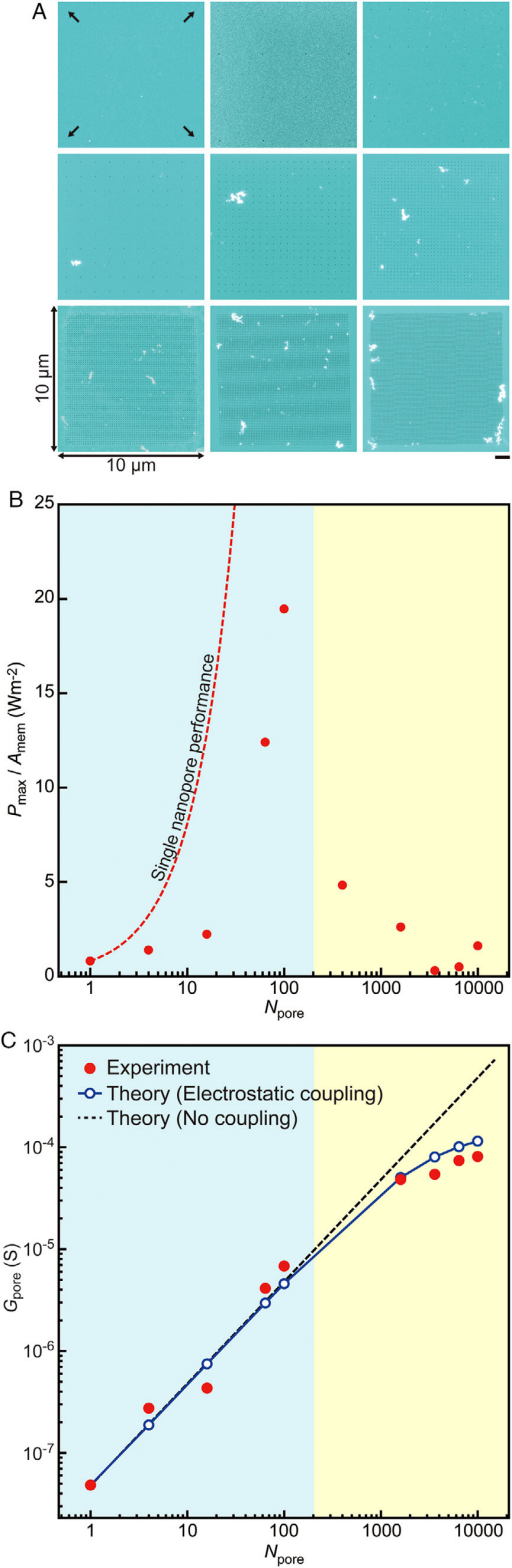
Multi‐nanopore osmotic power generators. (A) Scanning electron micrographs of two‐dimensional arrays of 20 nm‐sized nanopores in 40 nm‐thick SiN*_x_* membranes. The number of nanopores *N*_pore_ formed in the 10 µm × 10 µm areas varies from 4 to 10,000. Scale bar denotes 1 µm. (B) Maximum osmotic power density *P*_max_/*A*_mem_ of the multi‐nanopores. Dashed curve is the power output expected from the energy conversion efficiency of an individual 20 nm diameter pore without interpore interferences. (C) The open pore conductance *G*_pore_ of the multipores (red). The blue and black lines are the *N*_pore_ dependence of the nanopore conductance with and without electrostatic coupling at the access regions, respectively.

The above finding predicts varying osmotic energy conversion efficiencies in nanoporous membranes of different porosities. We, therefore, assessed the osmotic power outputs *P*_osm_ = *I*_osm_ *V*_dif_ under a 1000‐fold salinity difference for two‐dimensional arrays of 20 nm‐sized nanopores of varying numbers *N*_pore_ in the 10 µm × 10 µm regions of 40 nm‐thick SiN*_x_* membranes (Figure 4A, see also Figures S23–S31). While the increase in *N*_pore_ led to a nonlinear rise in the maximal power density^[^ ^30^ ^]^ *P*_max_/*A*_mem_ (=*P*_osm_/4*A*_mem_) due to the gain in *I*_osm_, where *A*_mem_ = 100 µm^2^ is the membrane area, it is revealed to drop significantly when the number exceeded 100 (corresponding to *d*_pp_ of 1000 nm) (Figure 4B). The situation was the same under smaller salinity gradients, where the maximal power density of 14 W m^−2^ was acquired at *c_trans_*/*c_cis_* = 200 (Figures S32 and S33). This is qualitatively the same as the case of the pair‐pore systems (Figure 3B,C) where the selectivity and osmotic power tended to decrease upon approaching the channels down to 100 nm. However, the changes in *S*_ion_ and *P*_osm_ are more prominent for the multipores suggesting a difference in the contributions of pore‐pore couplings.

Notably, the nanopores were separated by a distance exceeding 20 times their diameters at the point where the osmotic power exhibited a significant decline, a length considerably greater than the Dukhin length. Even under such a circumstance, a long‐range electrostatic coupling at the access regions is expected to cause a notable influence on the ion transport properties.^[^ ^22^, ^36^ ^]^ This gives rise to the *N*_pore_ (and *d*_pp_) dependence of the open pore conductance *G*_pore_ theoretically derived as

(1) $$G_{\mathrm{pore}}=N_{\mathrm{pore}}\sigma {\left\{\frac{4L_{\mathrm{mem}}}{\pi d_{\mathrm{pore}}^{2}}+\frac{1}{d_{\mathrm{pore}}}\left[1+\gamma \left(\frac{d_{\mathrm{pore}}}{2d_{\mathrm{pp}}}\right)\right]\right\}}^{-1}$$

with the solution conductivity *σ* and the coupling factor $\gamma =N_{\mathrm{pore}}^{0.5}$ for the two‐dimensional multi‐nanopore configurations.^[^ ^22^ ^]^ The fact that *G*_pore_ under *c_cis_* = *c_trans_* = 1.37 m becomes lower than *N*_pore_ *G*_single_ at around *N*_pore_ and *d*_pp_ of 100 and 500 nm, respectively, implies non‐negligible contributions of the electrostatic coupling in the low‐*P*_osm_ multipore membranes, where *G*_single_ = 128 nS is the single nanopore conductance (Figure 4C).

**FIGURE 5:**
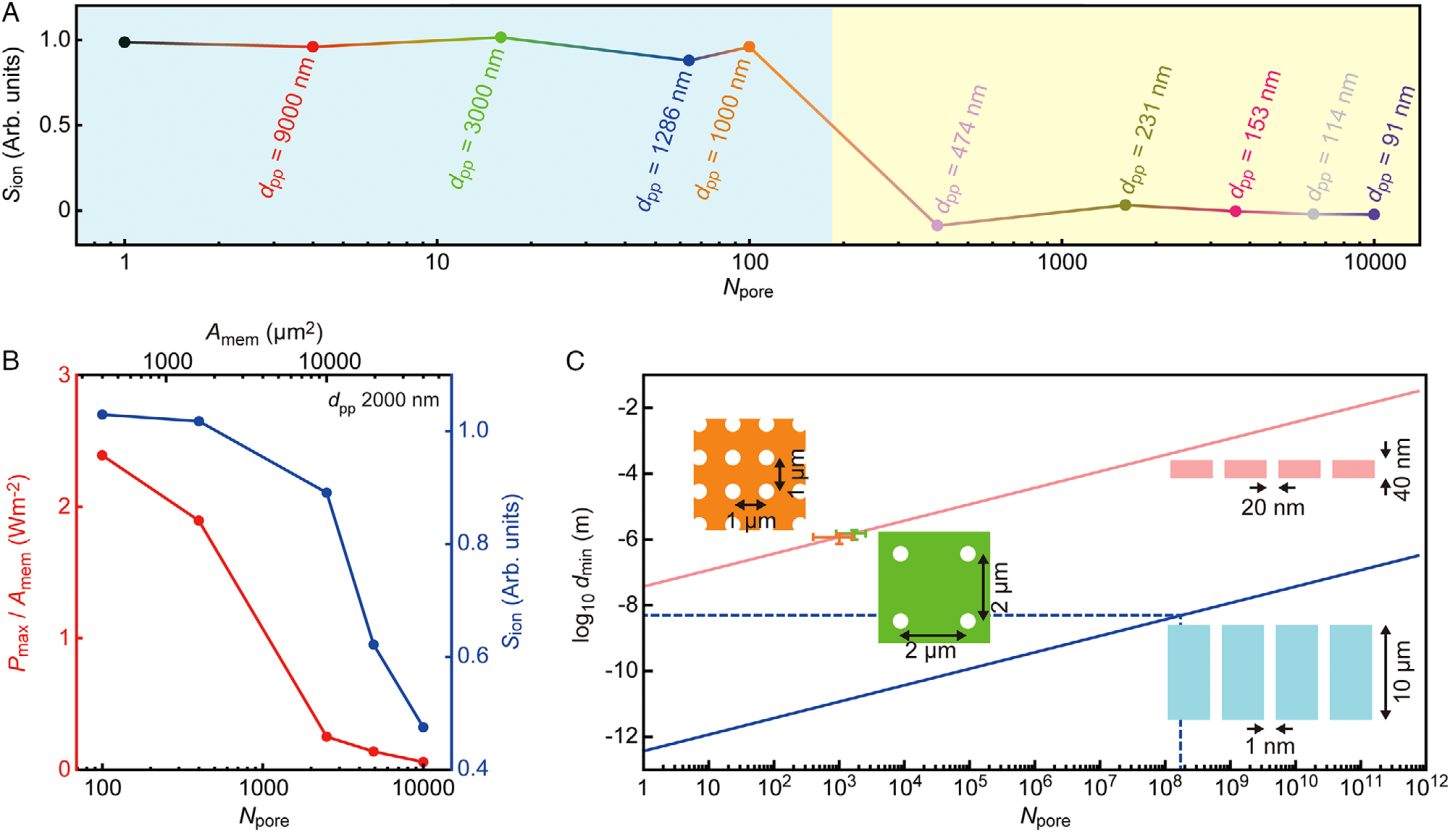
Electrostatic coupling‐derived osmotic power reduction in two‐dimensional arrays of multi‐nanopores. (A) Porosity dependence of *S*_ion_ in multipore membranes. Note the larger number of pores with shorter inter‐pore spacing of the membranes with higher porosity. A transition from permselective to non‐selective ion transport occurs when increasing the number of pores from 100 to 400. At this point, the pore‐pore spacing is also diminished from 1000 to 474 nm. (B) The osmotic power density *P*_max_/*A*_mem_ of multipore membranes at a constant porosity with *d*_pp_ = 2000 nm but different *N*_pore_. The power output drops rapidly upon increasing the number of pores. (C) The minimum‐possible inter‐pore spacing *d*_min_ for the pore‐pore interference‐free two‐dimensional multi‐nanopore arrays with the low‐ (pink) and high‐aspect‐ratio structures (blue). Orange and green bars denote the range of *d*_min_ and *N*_pore_ estimated from the data in (A) and (C), respectively. Blue dashed line points at the number of 1 nm‐sized nanochannels the 10 µm‐thick membrane can hold without significant effects of the pore‐pore electrostatic couplings.

A direct consequence of the electrostatic coupling is a weakened permselectivity as shown by the reduction in *S*_ion_ upon decreasing the grid spacing to below 500 nm (Figure 5A). Noticeably, the rectifying behaviors are also found to change appreciably by the membrane porosity (Figure S34). For example, *r*_rec_ at large *c_trans_*/*c_cis_* decreased at *N*_pore_ > 100 reflecting the undermined ion selectivity. Simultaneously, comparisons of the *r*_rec_ − *c_trans_*/*c_cis_* relationships with those obtained from the single channels (Figure 2D) indicated a growing influence of the electroosmotic mechanism with increasing the number density of the nanopores, which implied an ability of the electrostatic crosstalk at the access regions to virtually merge the closely‐packed multiple‐nanopores into a large conduit. All these results consistently denote a transition from permselective to non‐ion‐selective membranes via the electrostatic inter‐pore interactions.

As seen above, the electrostatic coupling‐derived entrance effects degrade the osmotic energy conversion efficiencies of multipore membranes. Although the situation is similar for the pair‐pore systems (Figure 3C), they demonstrate no such critical effects of the electrostatic coupling even under the narrow spacing below 500 nm. This may not be surprising for its effective size would be still smaller than *L*_D_ so as to persist strong permselectivity even when the two channels are electrically merged together via the electrostatic interference. Meanwhile, it also exemplifies the importance of not only the inter‐pore distance but also the number and coordination of the channels for controlling the electrostatic interactions.

It is worth testing to further shorten the interpore distance in the multipore structures to below 90 nm as we have seen positive effects of the pore‐pore coupling on *S*_ion_ and *P*_osm_ in the 30 nm‐spaced pair‐pores. Unfortunately, however, we were not able to examine it since the fringes of the SiN*_x_* channels became too narrow upon decreasing the interpore distance by increasing the porosity above 100 × 100 so that the entire structure broke down after the dry etching processes (Figure S35).

### 2.4 Scalability of osmotic power generation

In light of the analytical model,^[^ ^22^ ^]^ it becomes evident that long inter‐pore distance alone cannot guarantee the negligible influence of the electrostatic pore‐pore couplings on salt gradient‐mediated ion transport. The observation contrasts with previous notions suggesting that nanopores could be considered isolated by arranging their inter‐pore distance to be larger than the Debye screening length^[^ ^37^ ^]^ or the Dukhin length.^[^ ^26^ ^]^ To validate this finding, the osmotic power was measured for two‐dimensional nanopore arrays of 2000 nm spacing with *N*_pore_ up to 10000. Indeed, though *d*_pp_ >> *L*_D_, the sparse multipore membranes failed to maintain the permselectivity giving scarce osmotic power outputs with *N*_pore_ > 1000 (Figure 5B). More quantitatively, the theory predicts a prominent contribution of the entrance effect for the two‐dimensional multipore arrangements satisfying *γ* > *D*, where $D=L_{\mathrm{mem}}d_{\mathrm{pp}}/d_{\mathrm{pore}}^{2}$ is the non‐dimensional geometry factor.^[^ ^22^ ^]^ This is in fair agreement with the present results, where *S*_ion_ dropped when *αγ* exceeded *D* upon increasing *N*_pore_ as seen in Figures 4B and 5C (the coefficient *α* = 3.7 accounts for the influence of the salinity gradient not included in the theoretical model), thereby corroborating the predominant role of the electrostatic coupling at the access space on the reverse dialysis in multi‐nanopore membranes. Nevertheless, the precise mechanism linking selectivity loss to pore‐pore coupling remains unclear. In this regard, it is noticeable that the ion concentration polarization (ICP) becomes significant for high pore density systems, which would lead to the decrease in the apparent permselectivity as the pore‐pore distance reduces.^[^ ^19^ ^]^ However, it remains uncertain how the electrostatic coupling affects the ICP behaviors and whether it can be significant even for the 20 nm‐sized pores separated by micrometers. Further efforts should thus be devoted to elucidate how the electrostatic coupling impairs the ion selectivity of multipore membranes.

## 3 CONCLUSION

The results can be extended to multipores of different configurations in terms of the shortest inter‐pore distance $d_{\min}=\alpha \gamma d_{\mathrm{pore}}^{2}/L_{\mathrm{mem}}$ available without suffering from the coupling‐derived *S*_ion_ reduction (Figure 5C). In case of synthetic membranes such as anodic aluminum oxides^[^ ^38^ ^]^ and track‐etched polymer sheets,^[^ ^39^ ^]^ they normally contain single‐digit nanochannels of spacing shorter than 5 nm but lengths longer than 10 µm, which gives *D* of about 5000. While the present work anticipates weak electrostatic couplings in such a dense nanofluidic network due to the high length‐to‐diameter aspect ratio motifs, it still expects a pronounced entrance effect for the membranes larger than *L*_mem_ = 10 µm or equivalently 100 µm^2^. At the other extreme of self‐organized nanopore structures with ultrashort pore lengths such as those in metal‐^[^ ^16^ ^]^ and covalent‐organic frameworks,^[^ ^17^ ^]^ the electrostatic pore‐pore coupling would be almost inevitable. Meanwhile, we add to note that the size of the channels should be designed with respect to the salinity conditions involved to obtain low ionic resistance in the reservoirs with good ion selectivity.^[^ ^18^, ^21^ ^]^ These findings hold potential not only for the design of scalable multi‐nanopore membranes aimed at osmotic power generation and desalination but also for a better fundamental understanding of biological systems, where the ion channels and pumps in a single‐cell membrane synergistically work to implement various tasks from salt gradient regulations to action potential generation.

## 4 EXPERIMENTAL SECTION

### 4.1 Fabrication of lithographically‐defined multi‐nanopore membranes

A 4‐in. Si wafer of (100) orientation was covered with 50 nm‐thick SiN*_x_* layers by low‐pressure chemical vapor deposition. AZ5206E was spin‐coated on one side of the wafer and baked on a hot plate. Squares of 1 mm × 1 mm or 2 mm × 2 mm sizes were patterned by photolithography. After development, the exposed SiN*_x_* regions were etched by reactive ion etching with CHF_3_ etchant gas. This created SiN*_x_* windows that were used to obtain membranes by wet‐etching the Si layer in a heated KOH solution. The anisotropic etching formed trenches with 40 nm‐thick square membranes of 0.01 or 0.09 mm^2^ area at the bottom. The Si wafer was then cut into 30 mm × 30 mm square chips by dicer. On the membrane, ZEP520A (Zeon) was spin‐coated. After baking on a hot plate, one to 20,000 circles of various diameters (20 nm to 10 µm) and spacing (50 nm to 5 µm) were delineated by electron beam lithography (Elionix). The residual resist layer after development was used as a mask to form nanopore(s) by reactive ion etching with CHF_3_. The nanopore chip was immersed in *N,N*‐dimethylformamide overnight to dissolve the remaining resist followed by rinsing with ethanol and acetone. Finally, the chip was dried under nitrogen flow.

### 4.2 Preparation of polydimethylsiloxane flow cell

SU‐8 3000 (MICROCHEM) was spin‐coated on a 4‐in. Si wafer with 50 nm‐thick thermally‐grown SiO_2_ layers and pre‐baked on a hot plate. I‐shaped patterns were formed by photolithography, soft‐baking, and development in 2‐isopropanol. After hard baking, we obtained an SU‐8 mold for forming polydimethylsiloxane (PDMS) blocks with fluidic channels. This was done by first mixing the base and curing agent of Sylgard 184 (Dow) at 10 to 1 weight ratio in a disposable cup. It was then put in a desiccator and evacuated with a vacuum pump for 1 h to remove bubbles. The mixture solution was poured on the mold and heated at 90°C for 9 h in an oven for curing PDMS.

### 4.3 Bonding of PDMS flow cells

Two blocks of the PDMS on the SU‐8 mold were cut out with a knife. In each block, three holes were punched. After that, their surface as well as the nanopore chip were treated with oxygen plasma (FEMTO), which was‐then put together for eternal bonding.

### 4.4 Ionic current measurements

Salt solutions of various ion concentrations were prepared by diluting phosphate‐buffered saline of 1.37 m NaCl (Nippon Gene) with ultrapure water (Millipore). 1.37 m NaCl solution was poured into both the *cis* and *trans* sides of the nanopore chip via the three holes in each PDMS cell. To measure the ionic current through the pore membrane, Ag/AgCl rods were inserted into one of the holes in the PDMS blocks. The transmembrane voltage of *V*_b_ was added to the *trans*‐side of the Ag/AgCl and the output ionic current *I*_ion_ was measured through the electrode at the *cis* using a picoammeter/source unit (Keithlery 6487, Keithley) under a GPIB control (GPIB‐USB‐HS, National Instruments). In the measurements, *V*_b_ was scanned repeatedly from +1.1 to −1.1 V and −1.1 V to +1.1 V at a 5 mV step. After completing the data recording, the 1.37 m NaCl solution at the *cis* compartment was replaced with 0.69 m NaCl by pipetting the liquid several times through one of the holes in the PDMS flow cell, followed by another *I*_ion_—*V*_b_ characteristics measurements. These processes were repeated for diluter electrolyte buffers at the *cis* down to 1.37 mm NaCl. The data were used to calculate the average and standard deviation in *I*_ion_ at each *V*_b_. All the processes were performed under a program coded in Visual Basic.

### 4.5 Finite element analysis

Coupled Poisson–Nernst–Planck and Navier–Stokes equations were solved by MUMPS under steady‐state conditions to calculate the ion transport in nanopores using COMSOL Multiphysics 5.6. An axisymmetric model of a 1.5 µm‐sized pore in a 40 nm‐thick SiN*_x_* was used to deduce the electro‐osmotically‐driven ionic current rectification behavior under a 1000‐fold transmembrane salinity difference. On the other hand, 2D models of two 20 nm‐sized nanopores were employed to estimate the influence of inter‐pore distance on the spatial distributions of ion concentrations. See Figures S1 and S2 for the details including the models of the nanopore structures as well as the physical parameters (the surface potential, surface charge density of pore wall and membrane surface, ion concentrations, and pressure) and boundary conditions used for the simulations.

## CONFLICT OF INTEREST STATEMENT

The authors declare no conflicts of interest.

## Supporting information

Supporting Information

## Data Availability

The data that support the findings of this study are available from the corresponding author upon reasonable request.
