# Supplementary material for: Scalability of nanopore osmotic energy conversion
Source: Exploration (Beijing). 2024 Jan 8;4(2):20220110. doi: 10.1002/EXP.20220110 (PMC11022616; doi:10.1002/EXP.20220110)
Supplement: Supplementary file 1 — Supporting Information [file EXP2-4-20220110-s001.pdf]

Copyright WILEY-VCH Verlag GmbH & Co. KGaA, 69469 Weinheim, Germany, 2016.

Supporting Information

Scalability of nanopore osmotic energy conversion

Makusu Tsutsui, Wei-Lun Hsu, Kazumichi Yokota, Iat Wai Leong, Hirofumi Daiguji, and Tomoji Kawai

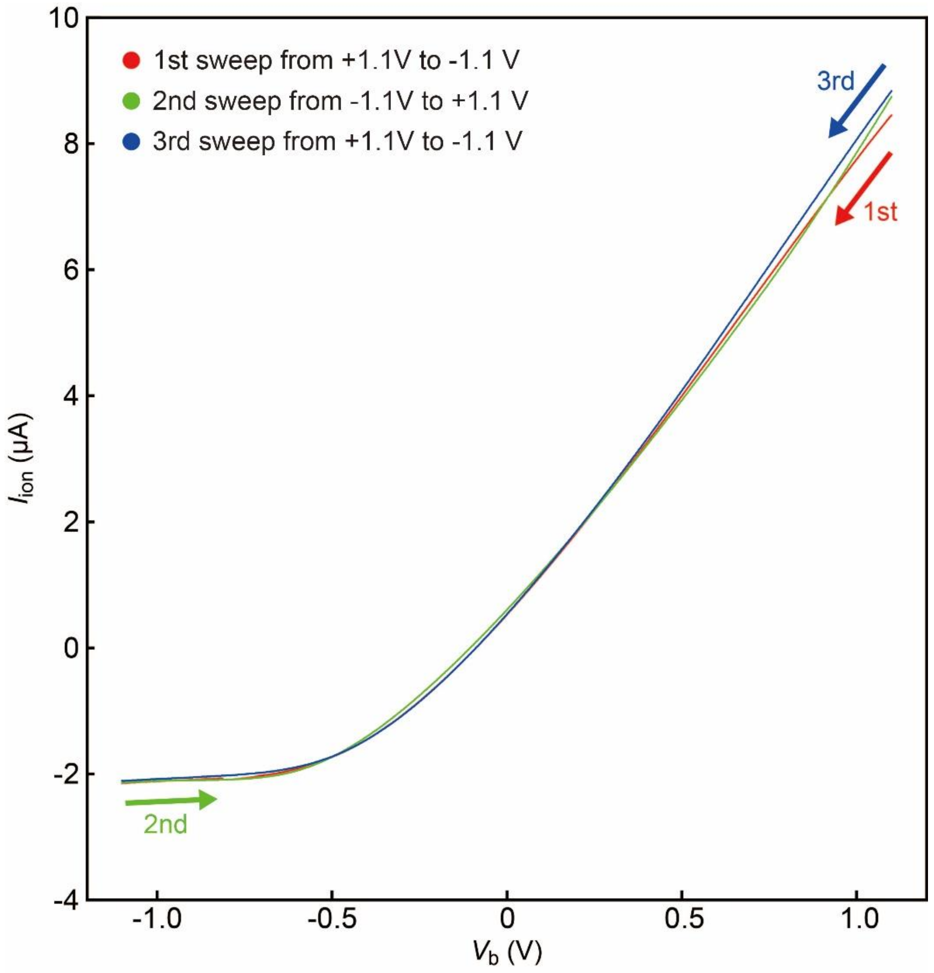

Figure S1. Ionic current (I_{ion}) versus transmembrane voltage (V_b) characteristics of a two-dimensional array of 20 nm diameter nanopores in a 40 nm-thick SiN_x membrane under a salinity difference of c_{cis} = 68.5 mM NaCl and c_{tans} = 1370 mM NaCl. The measurements were performed by scanning V_b several times in a range from +1 V to -1 V. The average and the standard deviation were calculated from the data, which were displayed in the following figures for all the cases tested including the different pore diameters, ion concentration gradients, inter-pore distance, and the number of pores. Only average data are shown in the main text for the sake of clarity. Arrows indicate the direction of the voltage sweeps.

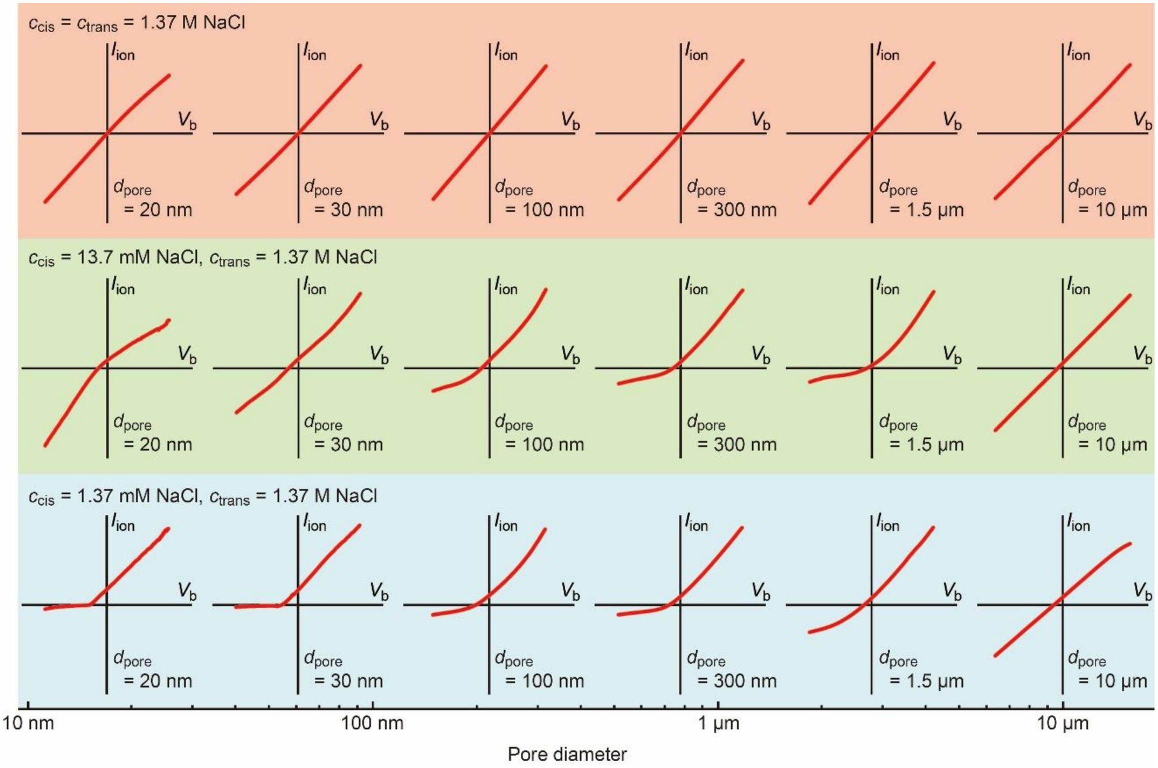

Figure S2. Salt gradient-mediated ionic current (I_{ion}) versus transmembrane voltage (V_b) characteristics of a cylindrical channel in a 40 nm-thick SiN_x membrane. The redox potential difference (V_{ele}) is subtracted from V_b (Table S1). Red, green, and skyblue colors denote the *cis*-to-*trans* ion concentration ratio (c_{trans}/c_{cis}). d_{pore} is the diameter of the pores. The curves are almost linear and cross zero current at zero voltage when there are no salt gradients irrespective of d_{pore} (red). On the other hand, smaller pores tend to show stronger rectifying behaviors with 1000-fold salt gradients (skyblue). Another distinct feature can be seen at the intermediate salt concentration difference, where the polarity of the diode characteristics is inverted in the case of a 20 nm-sized nanopore (green). In addition to the curvatures, larger negative intersects at zero current are found for smaller pores under higher ion concentration ratio c_{trans}/c_{cis} suggestive of larger diffusion potential difference induced by the salt gradient-mediated ion transport in conduits of stronger permselectivities.

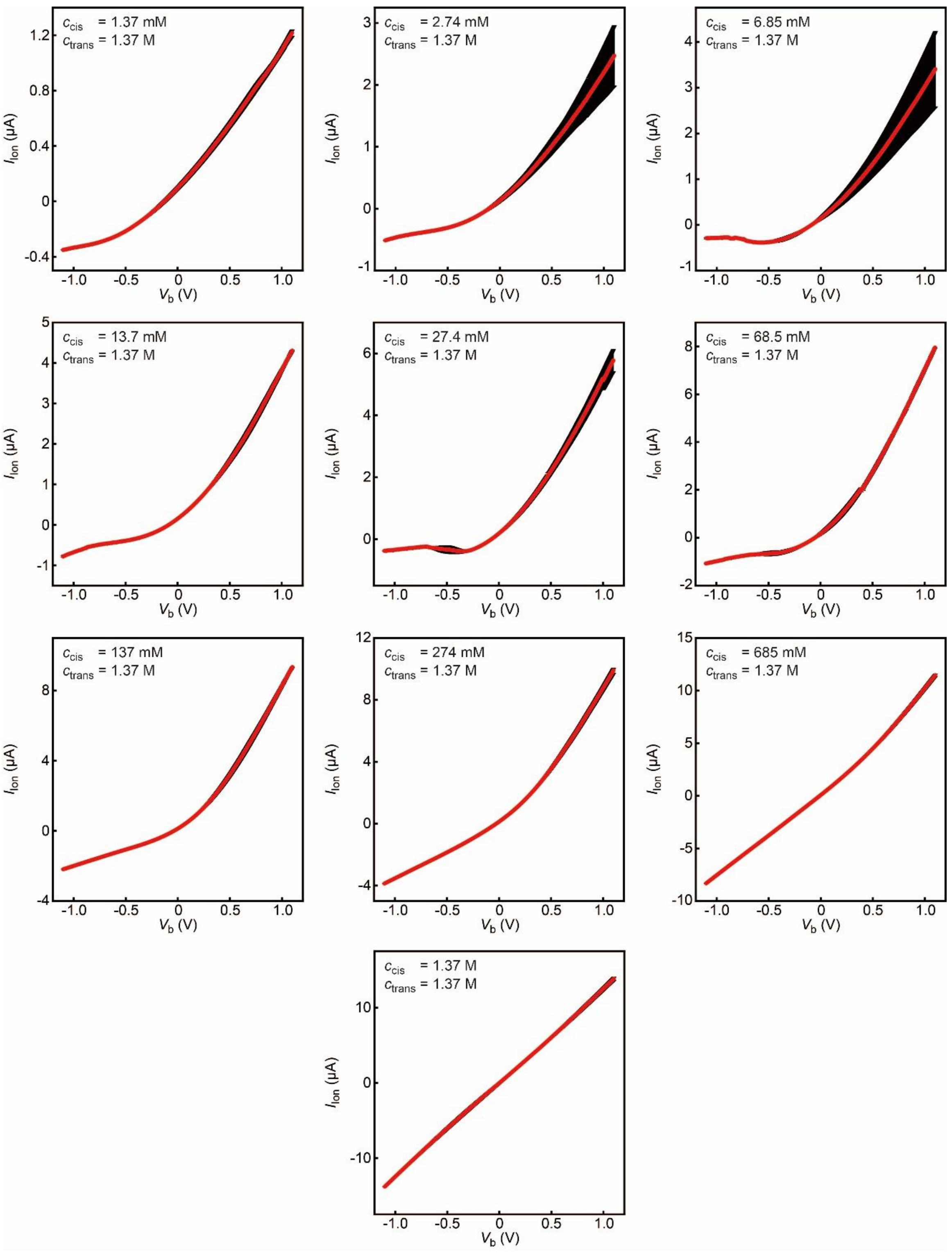

Figure S3. Ionic current (I_{ion}) versus transmembrane voltage (V_b) characteristics of 1.5 μm-sized micropore in a 40 nm-thick SiN_x membrane under various salt gradients. The ion concentration at *cis* (c_{cis}) and *trans* (c_{trans}) denote the salinity difference across the membranes. Red plots are the average I_{ion} estimated from the data obtained by scanning V_b from +1 to -1 V and -1 to +1 V. Error bars show the standard deviations.

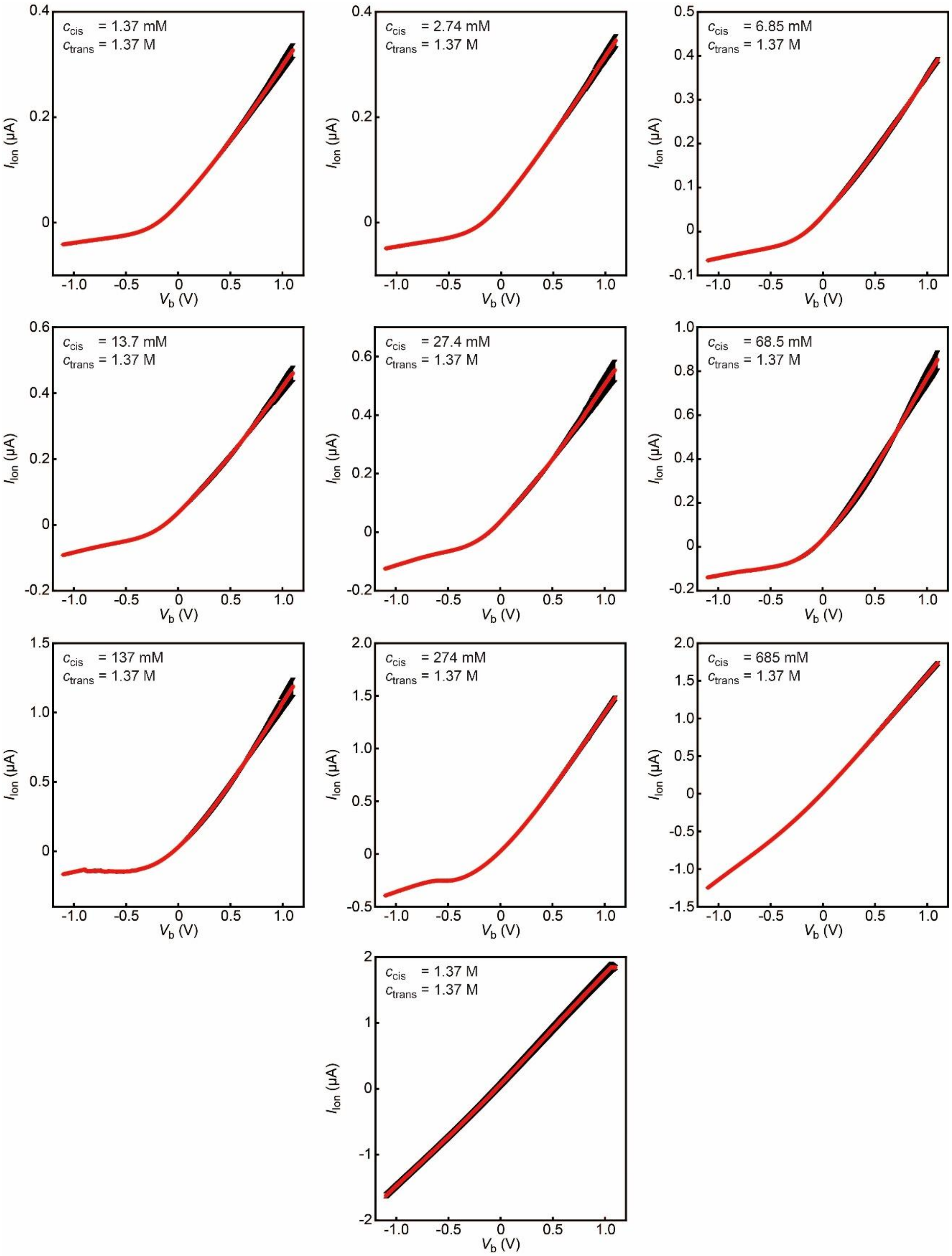

Figure S4. Ionic current (I_{ion}) versus transmembrane voltage (V_b) characteristics of 300 nm-sized pore in a 40 nm-thick SiN_x membrane under various salt gradients. The ion concentration at *cis* (c_{cis}) and *trans* (c_{trans}) denote the salinity difference across the membranes. Red plots are the average I_{ion} estimated from the data obtained by scanning V_b from +1 to -1 V and -1 to +1 V. Error bars show the standard deviations.

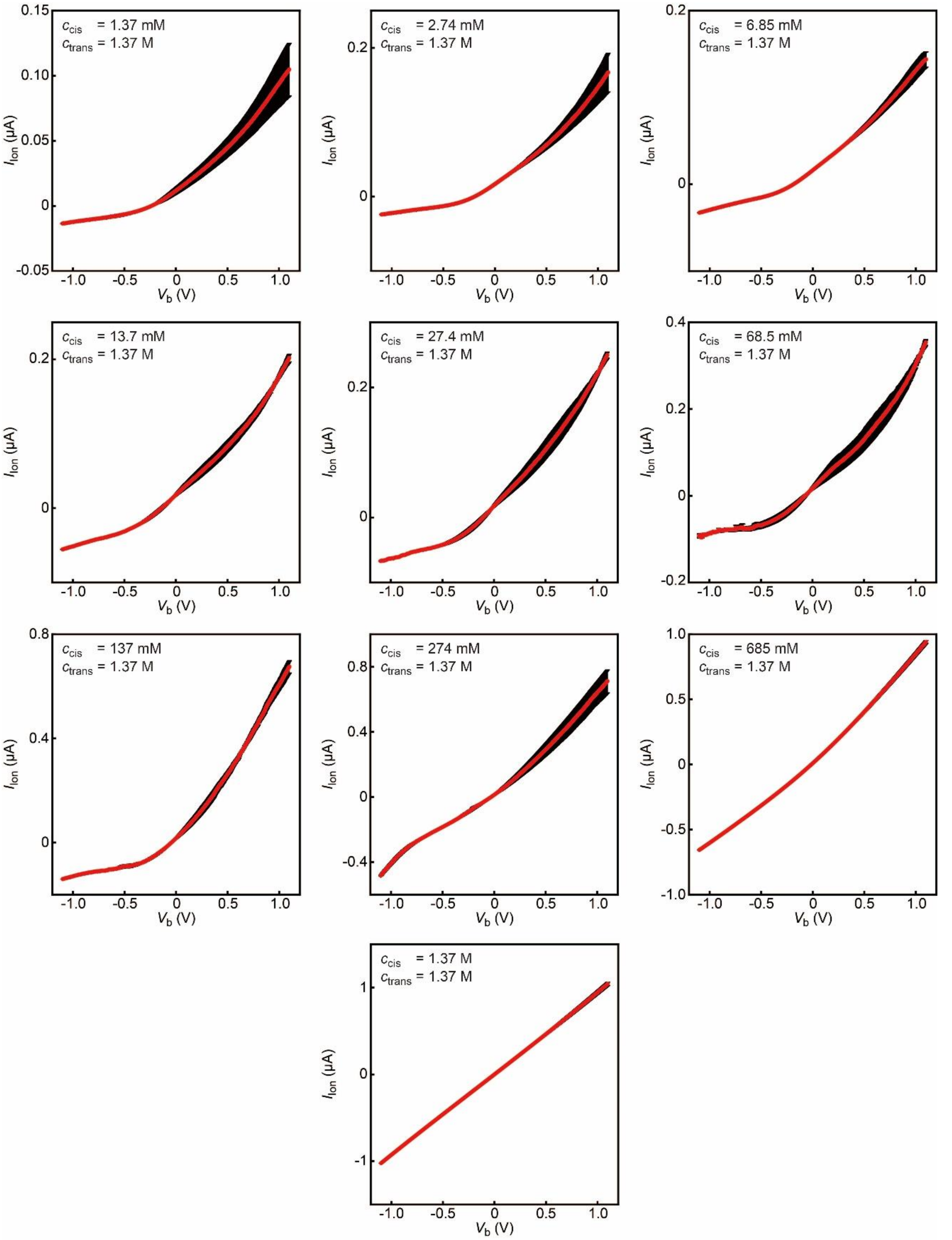

Figure S5. Ionic current (I_{ion}) versus transmembrane voltage (V_b) characteristics of 100 nm-sized nanopore in a 40 nm-thick SiN_x membrane under various salt gradients. The ion concentration at *cis* (c_{cis}) and *trans* (c_{trans}) denote the salinity difference across the membranes. Red plots are the average I_{ion} estimated from the data obtained by scanning V_b from +1 to -1 V and -1 to +1 V. Error bars show the standard deviations.

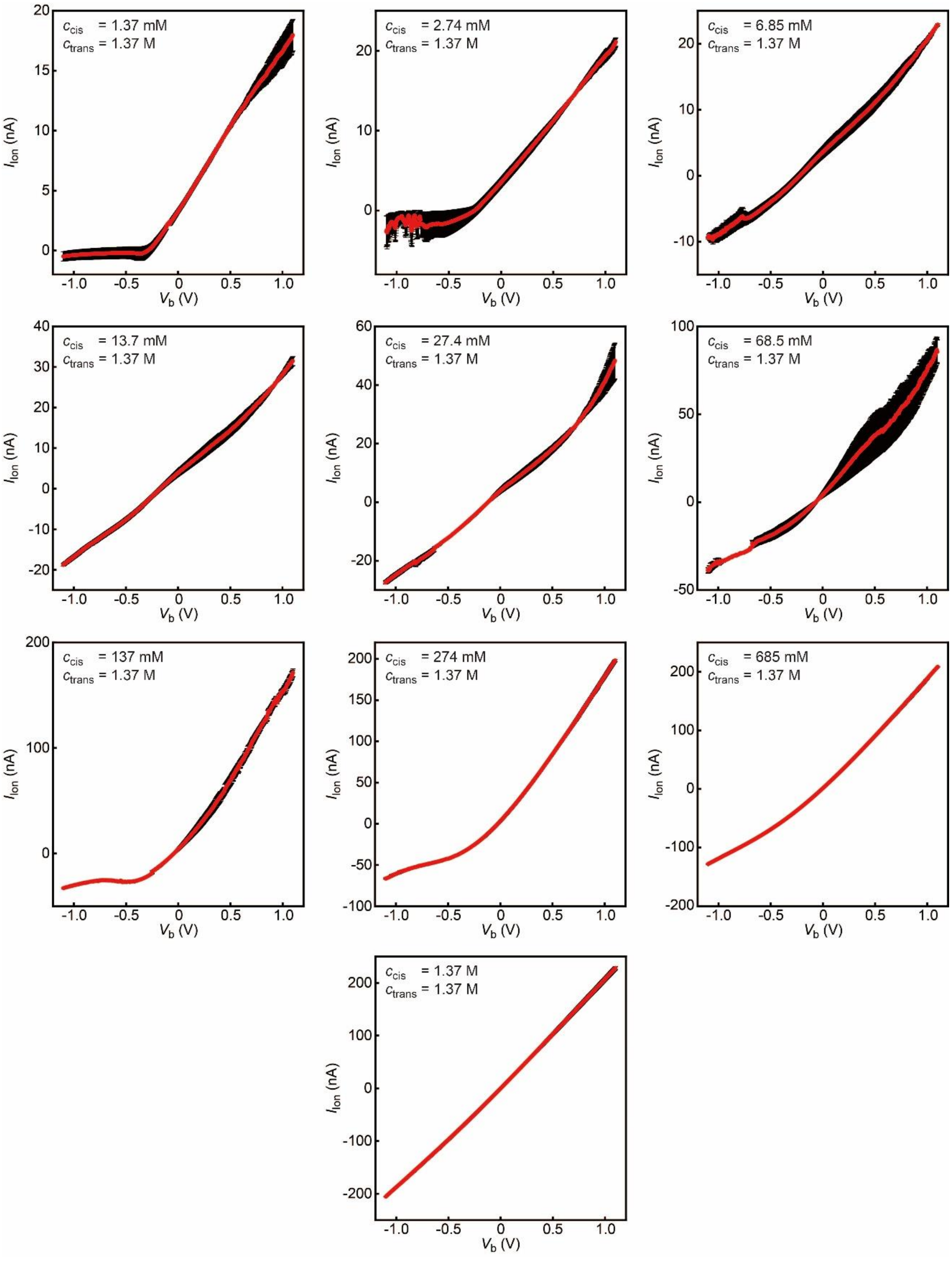

Figure S6. Ionic current (I_{ion}) versus transmembrane voltage (V_b) characteristics of 30 nm-sized nanopore in a 40 nm-thick SiN_x membrane under various salt gradients. The ion concentration at *cis* (c_{cis}) and *trans* (c_{trans}) denote the salinity difference across the membranes. Red plots are the average I_{ion} estimated from the data obtained by scanning V_b from +1 to -1 V and -1 to +1 V. Error bars show the standard deviations.

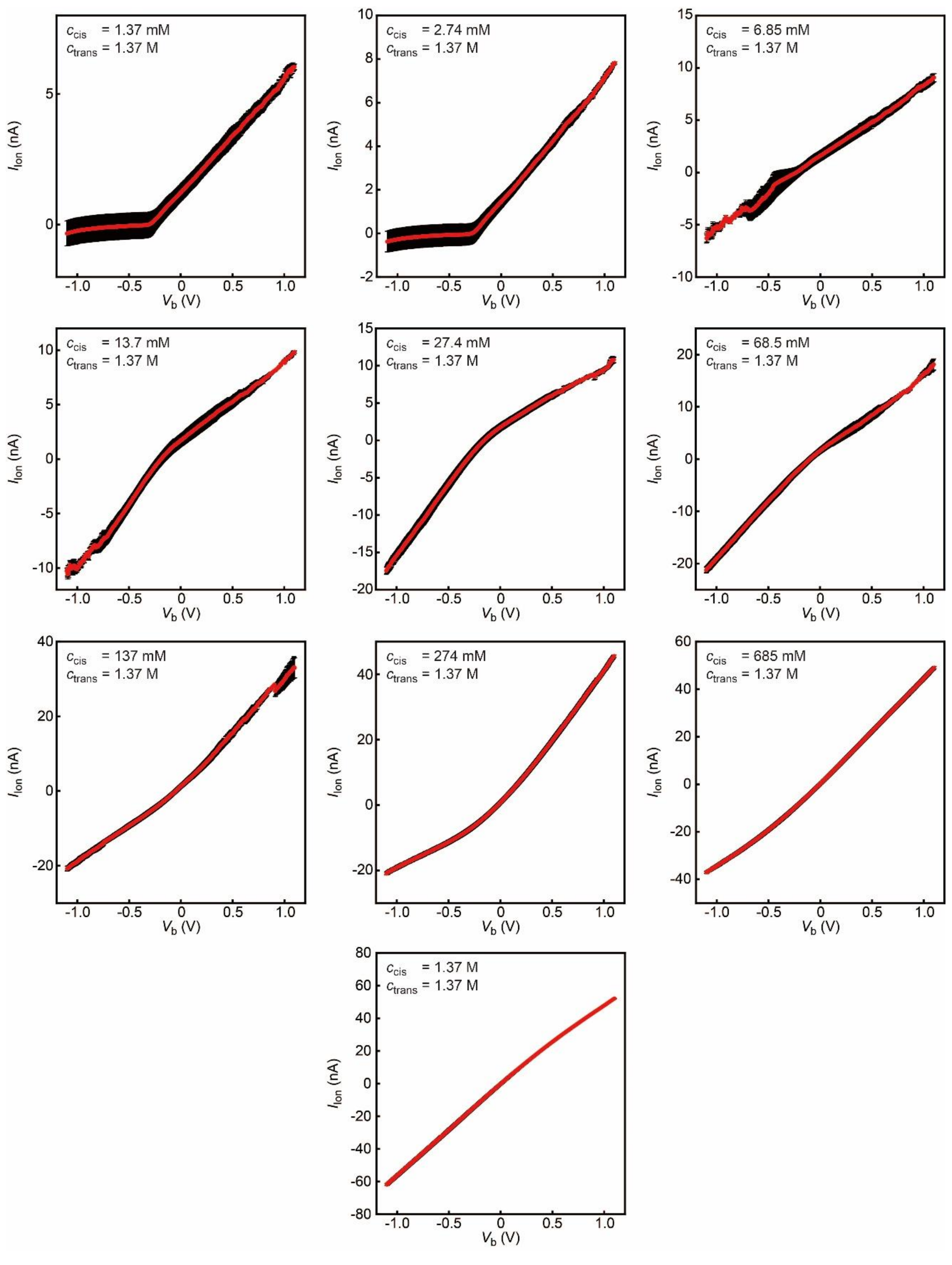

Figure S7. Ionic current (I_{ion}) versus transmembrane voltage (V_b) characteristics of 20 nm-sized nanopore in a 40 nm-thick SiN_x membrane under various salt gradients. The ion concentration at *cis* (c_{cis}) and *trans* (c_{trans}) denote the salinity difference across the membranes. Red plots are the average I_{ion} estimated from the data obtained by scanning V_b from +1 to -1 V and -1 to +1 V. Error bars show the standard deviations.

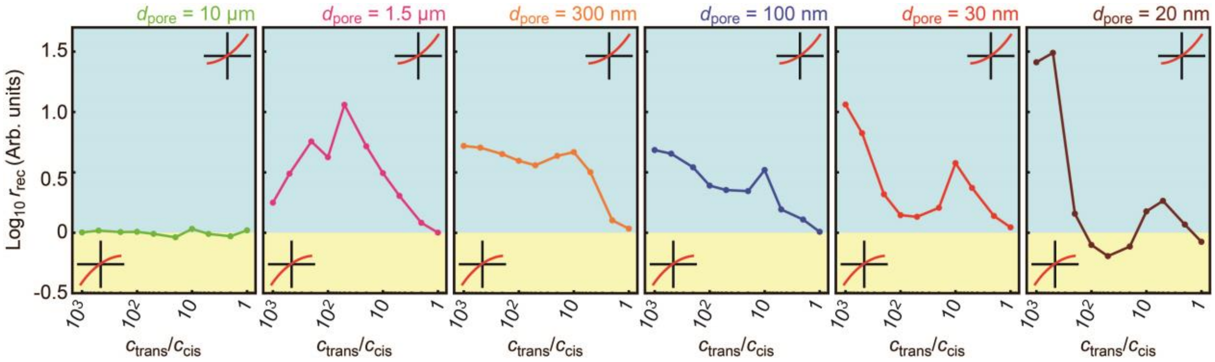

Figure S8. Ionic current rectification in a single pore under salinity gradients. The ionic current rectification ratio r_{rec} at ±0.8 V of the I_{ion} – V_b curves recorded for single pores of diameter d_{pore} (after subtracting V_{ele} from the data) under various salt concentration differences noted by c_{trans}/c_{cis}. Note that r_{rec} is shown in logarithmic scale so that the polarity of the rectifying behavior can be seen by its sign as depicted by the blue and yellow regions as well as the insets describing the actual rectification directions.

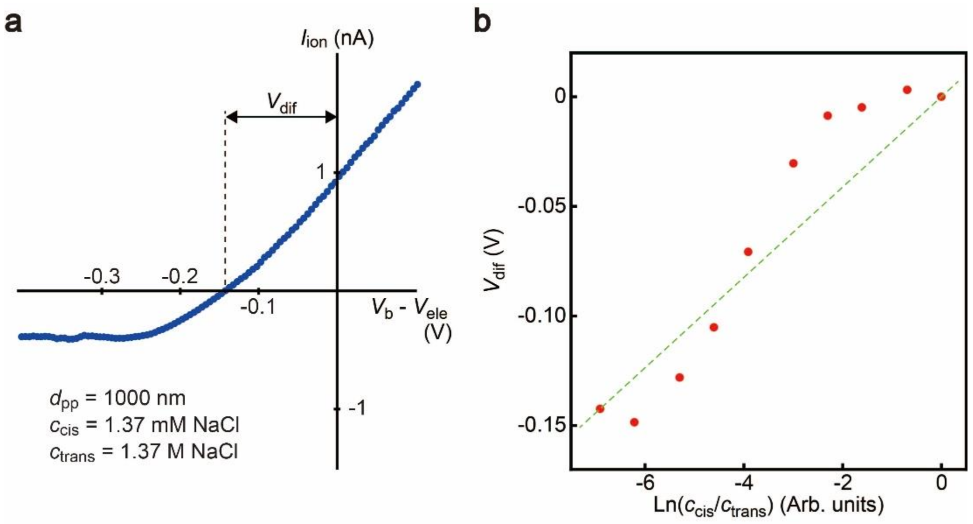

Figure S9. Estimation of ion selectivity in nanopores. a, The ionic current (I_{ion}) versus transmembrane voltage (V_b) characteristics of two 20 nm-sized pores (inter-pore distance d_{pp} = 1000 nm) in a 40 nm-thick SiN_x membrane with a 1000-fold *cis*-to-*trans* salt concentration difference. The redox potential V_{ele} was subtracted from V_b. The intersect at zero current defines the diffusion voltage V_{dif} that represents the degree of permselectivity as $V_{dif} = S_{ion}(k_B T/e)\ln(c_{cis}/c_{trans})$, where S_{ion} is the selectivity factor denoting perfect cation-selective and non-selective transport in the pore by S_{ion} = 1 and 0, respectively. **b,** V_{dif} plotted as a function of the logarithmic salt concentration ratio $\text{Ln}(c_{cis}/c_{trans})$. The green dashed line is a linear fit, whose slope is used to calculate S_{ion}.

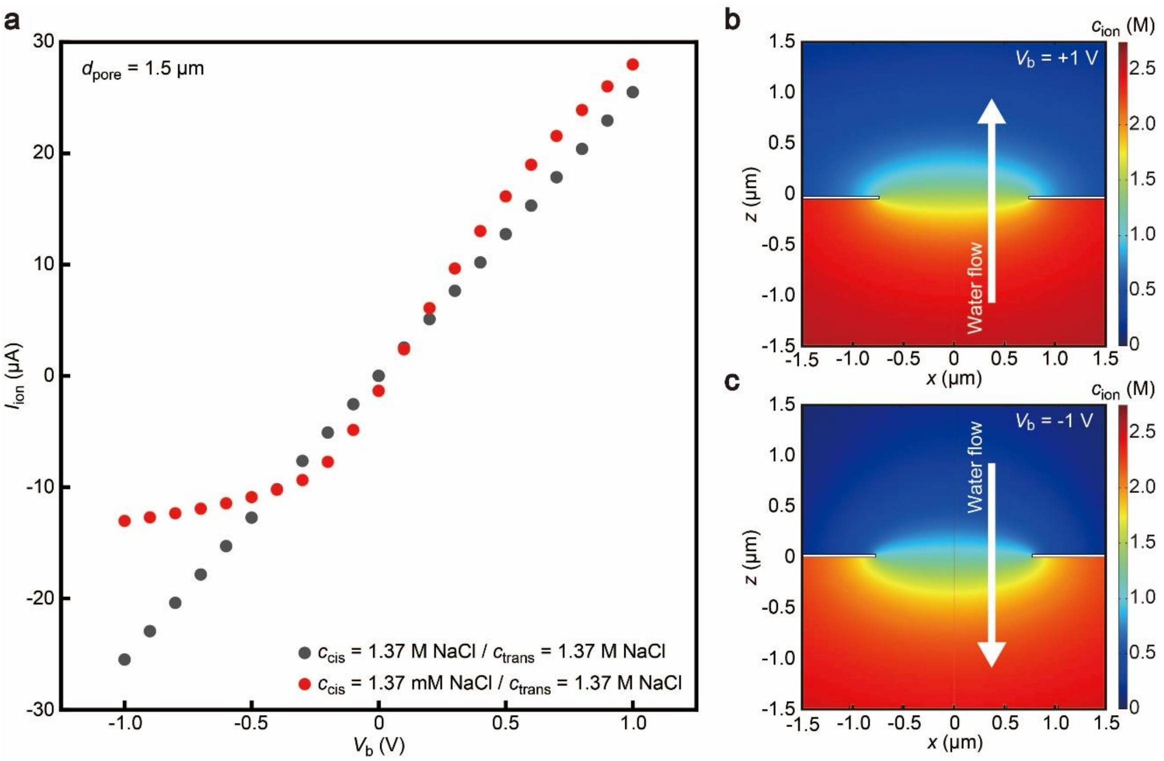

Figure S10. Theoretical estimations of ion transport in a 1.5 μm-sized pore in a 40 nm-thick SiN_x membrane under 1000-fold salt concentration difference at the *cis* and *trans* chambers. a, The ionic current (I_{ion}) versus transmembrane voltage (V_b) characteristics obtained by solving Poisson-Nernst-Planck and Navier-Stokes equations in a framework of a finite element method. The salt concentration at trans (c_{trans}) was set to 1.37 M NaCl while that at the *cis* (c_{cis}) was either 1.37 M (grey) or 1.37 mM (red). **b-c,** The ion concentration distributions around the micropore under V_b = +1 (b) and -1 V (c). Arrows indicate the direction of the water flow induced by electroosmosis due to the negative native charges on the SiN_x wall surface. The hydrodynamic flow pushes the high- (low-) concentration electrolyte solution into the pore thus lowering (raising) the pore resistance in a V_b-dependent manner to induce the rectifying behavior shown in (a).

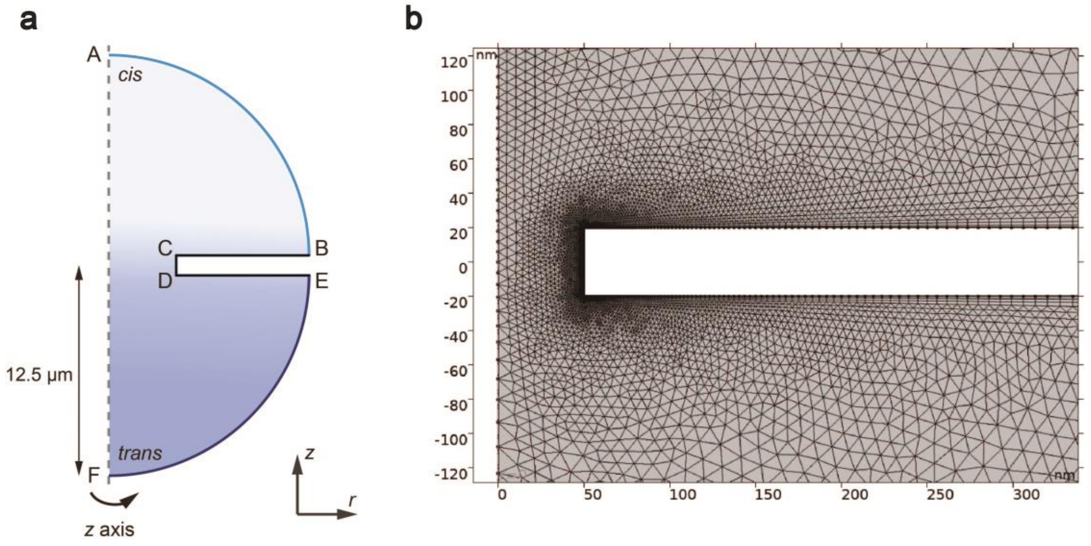

c

Boundaries	AC/ DC	Creeping fluid flow	Transport of diluted species
AF	Axisymmetry		
AB	Grounded ($\Phi = 0$)	Bulk concentration ($c = c_{cis}$)	No slip ($v = 0$)
EF	Applied terminal bias ($\Phi = V_b$)	Bulk concentration ($c = c_{trans}$)	
BC, CD, DE	Constant surface charge density (σ_{mem})	Zeta potential (ζ)	Ion impenetrable ($nN_i = 0$)

Figure S11. Model and conditions used for simulations of the electroosmotically-drived ionic current rectification in 1.5 μm-sized pore in a 40 nm-thick SiN_x. a-b, Geometry of the pair-pore system (a, not to scale) and the actual model with meshes (b). **c,** Boundary conditions for the regions A through M defined in (a). Φ, σ, c, n, N_i, μ_{EO}, p, and v are the surface potential, surface charge density of the pore wall and membrane surface, ion concentration, normal vector, ion flux, electroosmotic mobility, pressure, and fluid velocity, respectively. Zeta potential ζ is deduced from σ_{mem} = -15 mCm^{-2} by Graham equation.[S1]

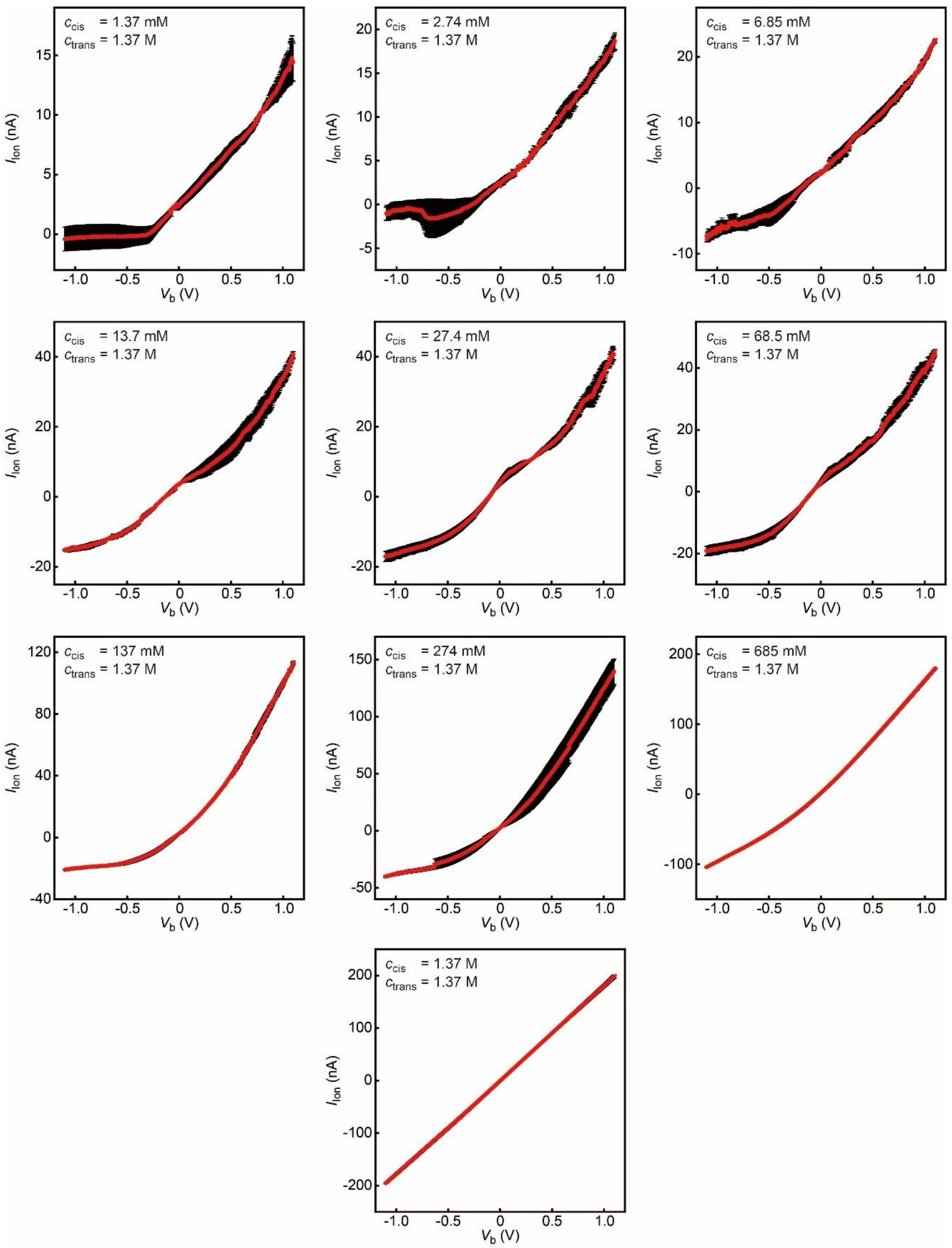

Figure S12. Ionic current (I_{ion}) versus transmembrane voltage (V_b) characteristics of two 20 nm-sized nanopores separated by 5000 nm in a 40 nm-thick SiN_x membrane under various salt gradients. The ion concentration at *cis* (c_{cis}) and *trans* (c_{trans}) denote the salinity difference across the membranes. Red plots are the average I_{ion} estimated from the data obtained by scanning V_b from +1 to -1 V and -1 to +1 V. Error bars show the standard deviations.

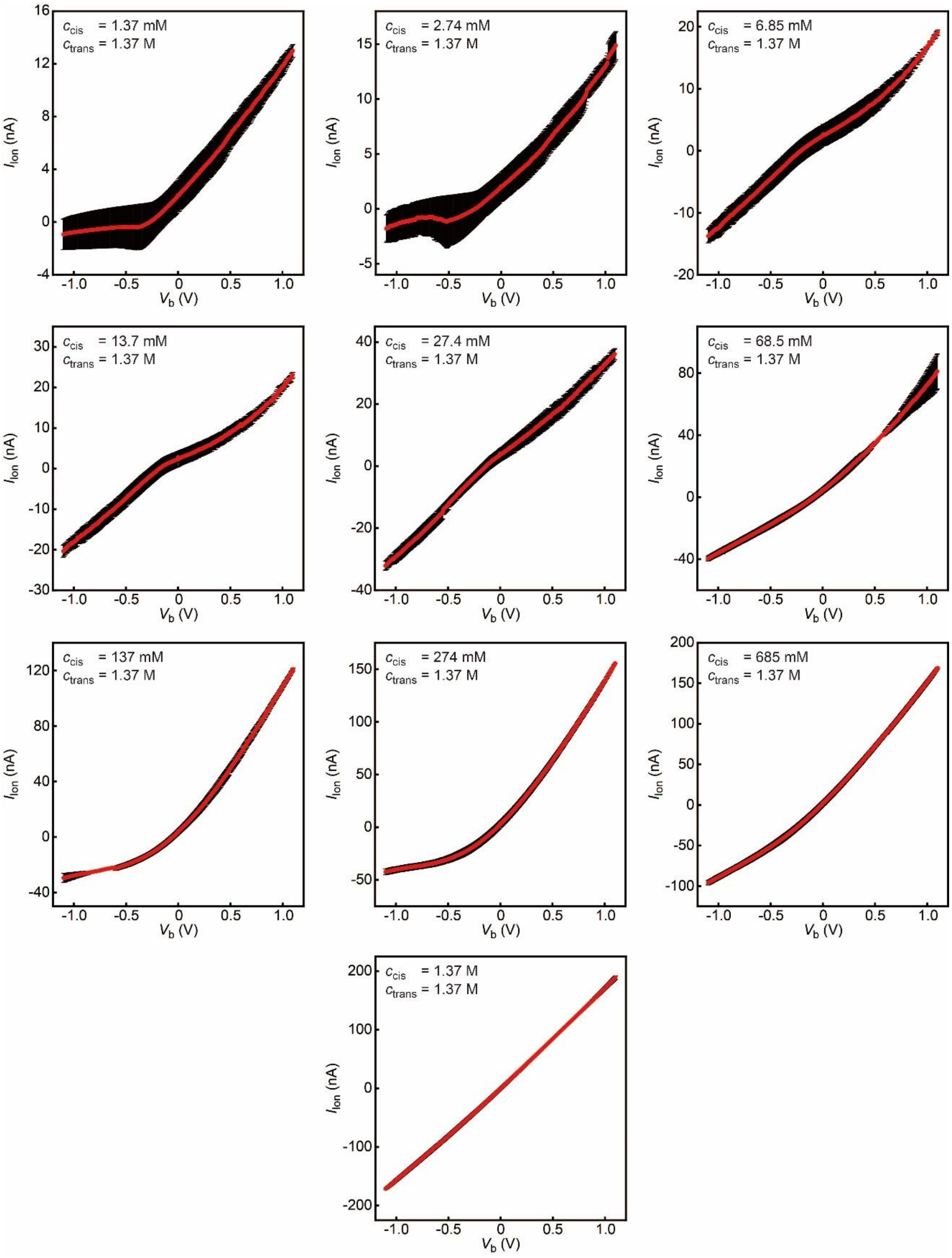

Figure S13. Ionic current (I_{ion}) versus transmembrane voltage (V_b) characteristics of two 20 nm-sized nanopores separated by 1000 nm in a 40 nm-thick SiN_x membrane under various salt gradients. The ion concentration at *cis* (c_{cis}) and *trans* (c_{trans}) denote the salinity difference across the membranes. Red plots are the average I_{ion} estimated from the data obtained by scanning V_b from +1 to -1 V and -1 to +1 V. Error bars show the standard deviations.

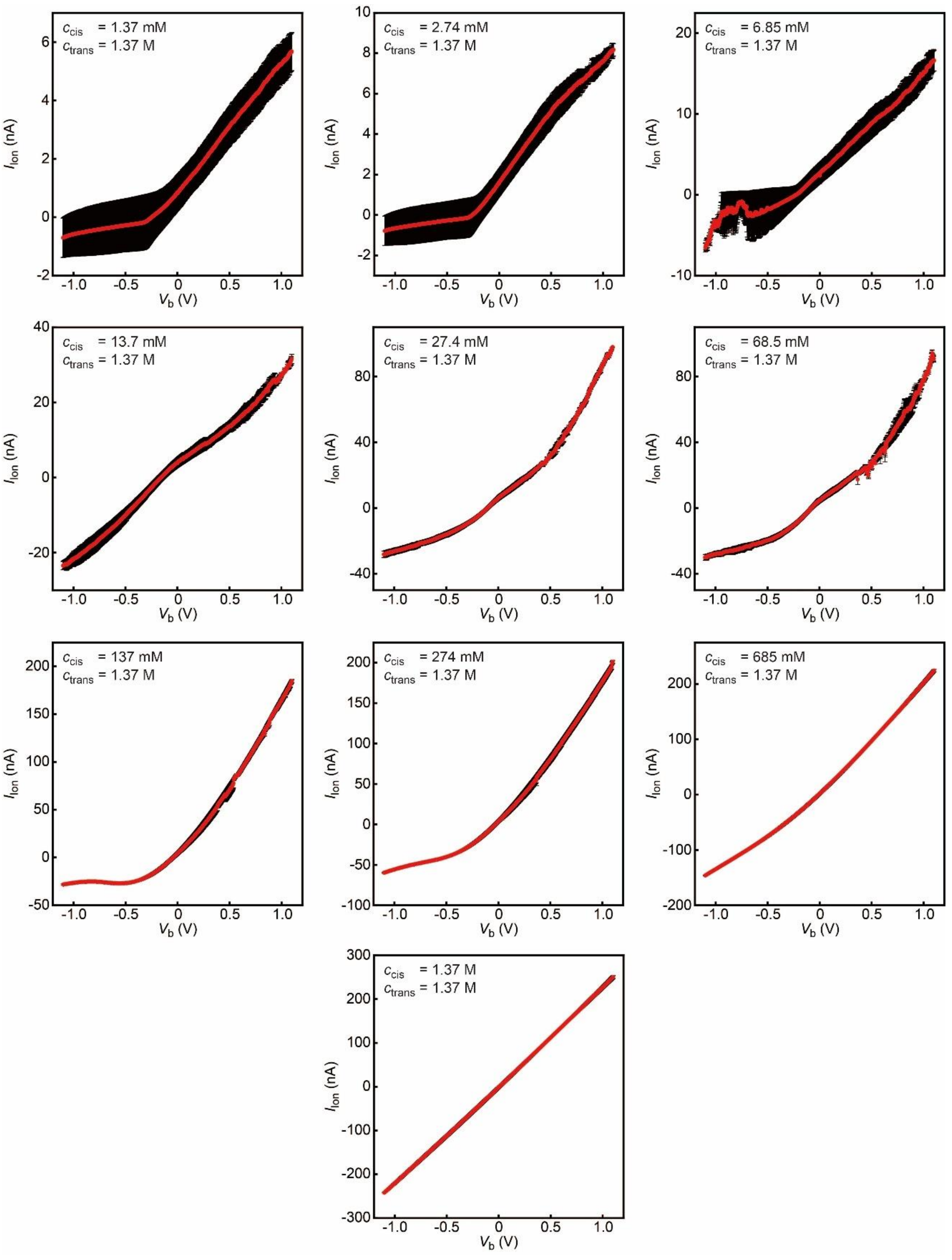

Figure S14. Ionic current (I_{ion}) versus transmembrane voltage (V_b) characteristics of two 20 nm-sized nanopores separated by 300 nm in a 40 nm-thick SiN_x membrane under various salt gradients. The ion concentration at *cis* (c_{cis}) and *trans* (c_{trans}) denote the salinity difference across the membranes. Red plots are the average I_{ion} estimated from the data obtained by scanning V_b from +1 to -1 V and -1 to +1 V. Error bars show the standard deviations.

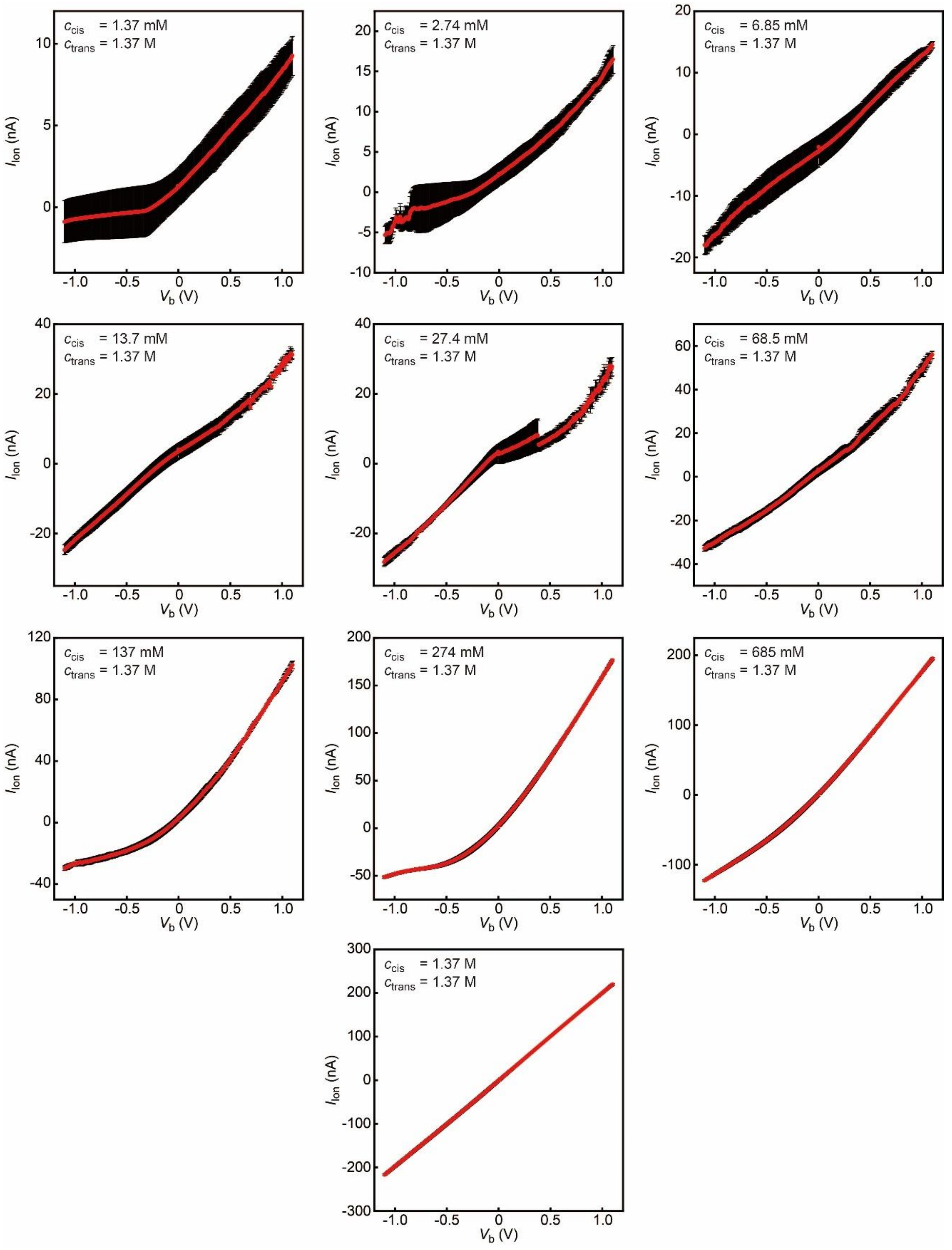

Figure S15. Ionic current (I_{ion}) versus transmembrane voltage (V_b) characteristics of two 20 nm-sized nanopores separated by 100 nm in a 40 nm-thick SiN_x membrane under various salt gradients. The ion concentration at *cis* (c_{cis}) and *trans* (c_{trans}) denote the salinity difference across the membranes. Red plots are the average I_{ion} estimated from the data obtained by scanning V_b from +1 to -1 V and -1 to +1 V. Error bars show the standard deviations.

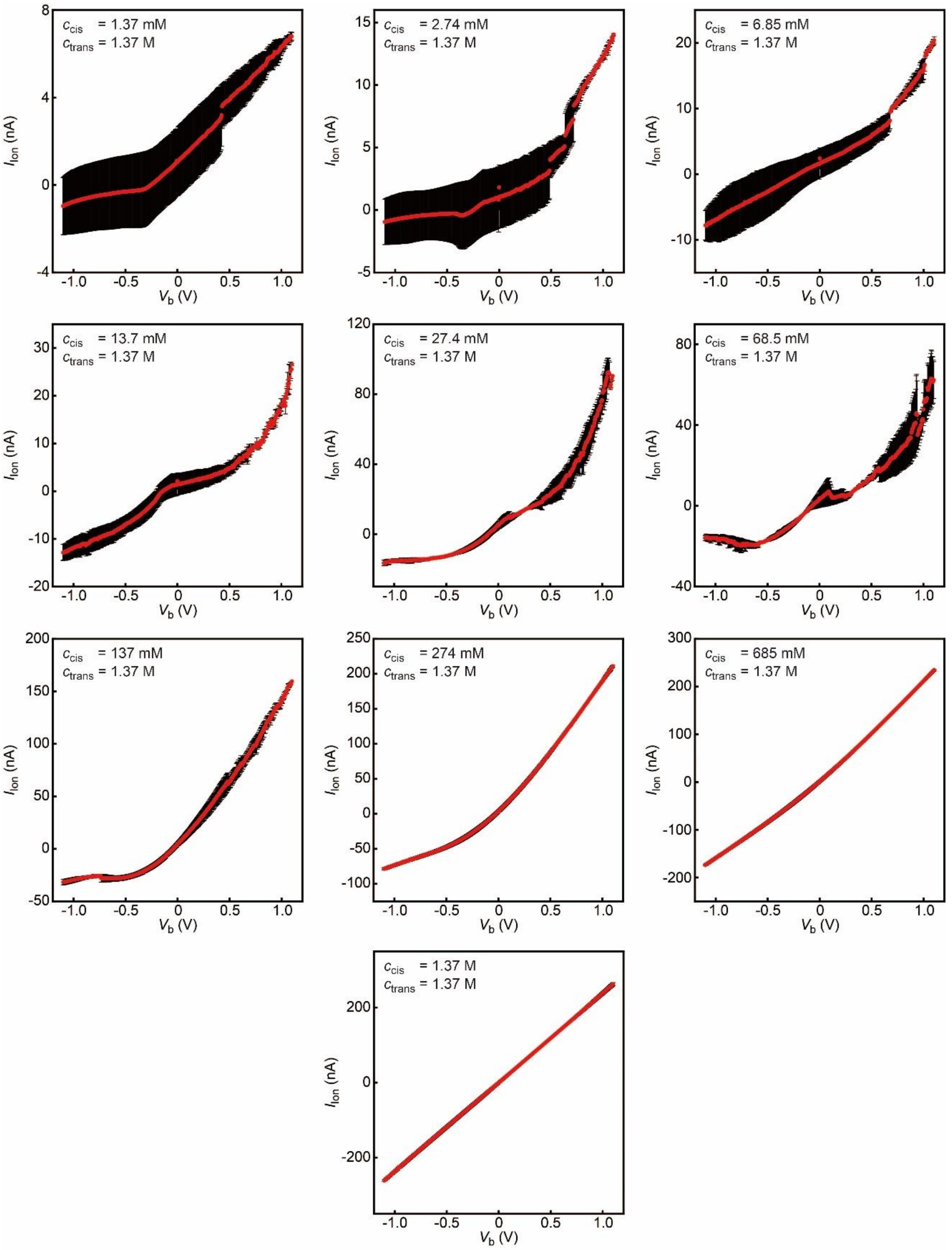

Figure S16. Ionic current (I_{ion}) versus transmembrane voltage (V_b) characteristics of two 20 nm-sized nanopores separated by 80 nm in a 40 nm-thick SiN_x membrane under various salt gradients. The ion concentration at *cis* (c_{cis}) and *trans* (c_{trans}) denote the salinity difference across the membranes. Red plots are the average I_{ion} estimated from the data obtained by scanning V_b from +1 to -1 V and -1 to +1 V. Error bars show the standard deviations.

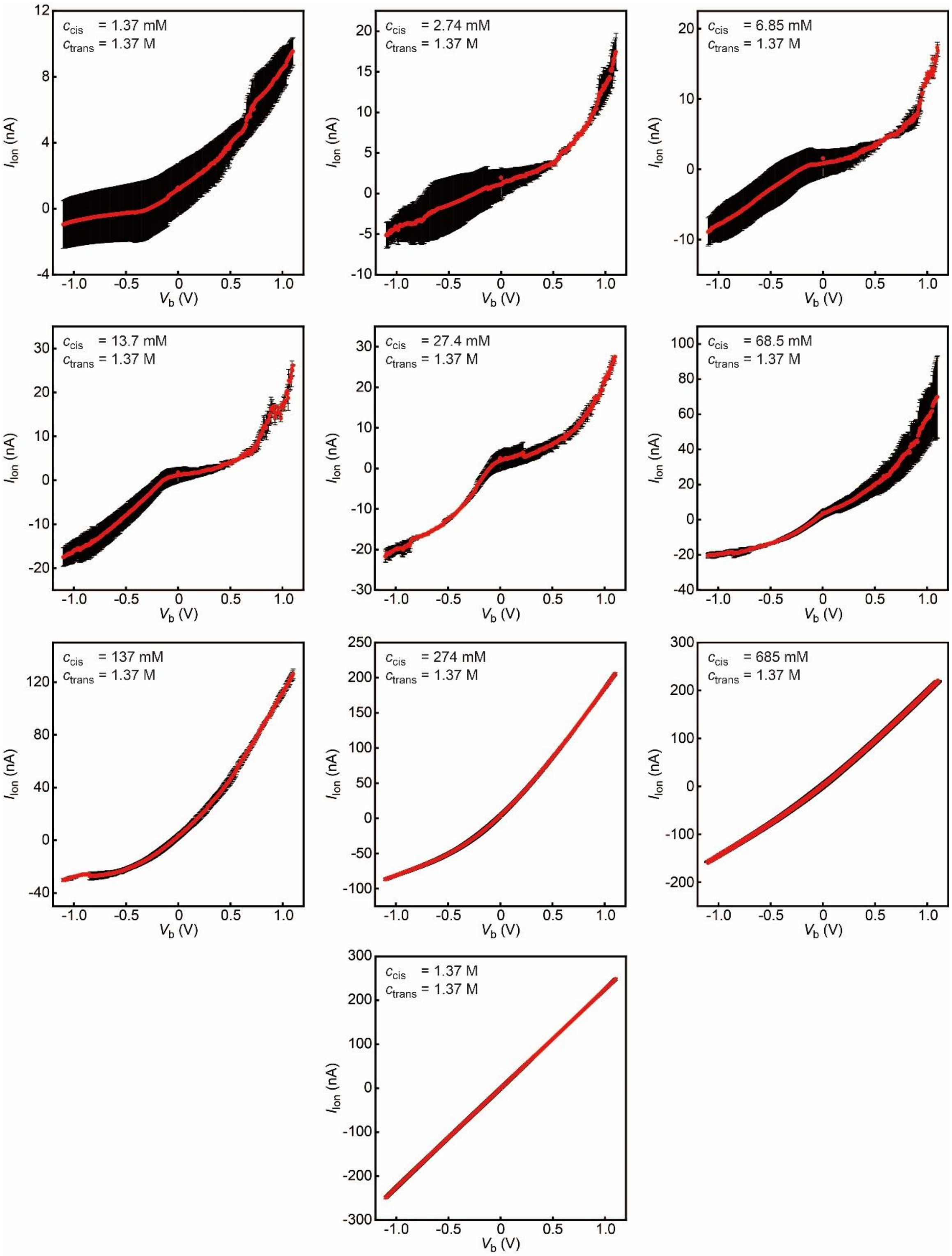

Figure S17. Ionic current (I_{ion}) versus transmembrane voltage (V_b) characteristics of two 20 nm-sized nanopores separated by 60 nm in a 40 nm-thick SiN_x membrane under various salt gradients. The ion concentration at *cis* (c_{cis}) and *trans* (c_{trans}) denote the salinity difference across the membranes. Red plots are the average I_{ion} estimated from the data obtained by scanning V_b from +1 to -1 V and -1 to +1 V. Error bars show the standard deviations.

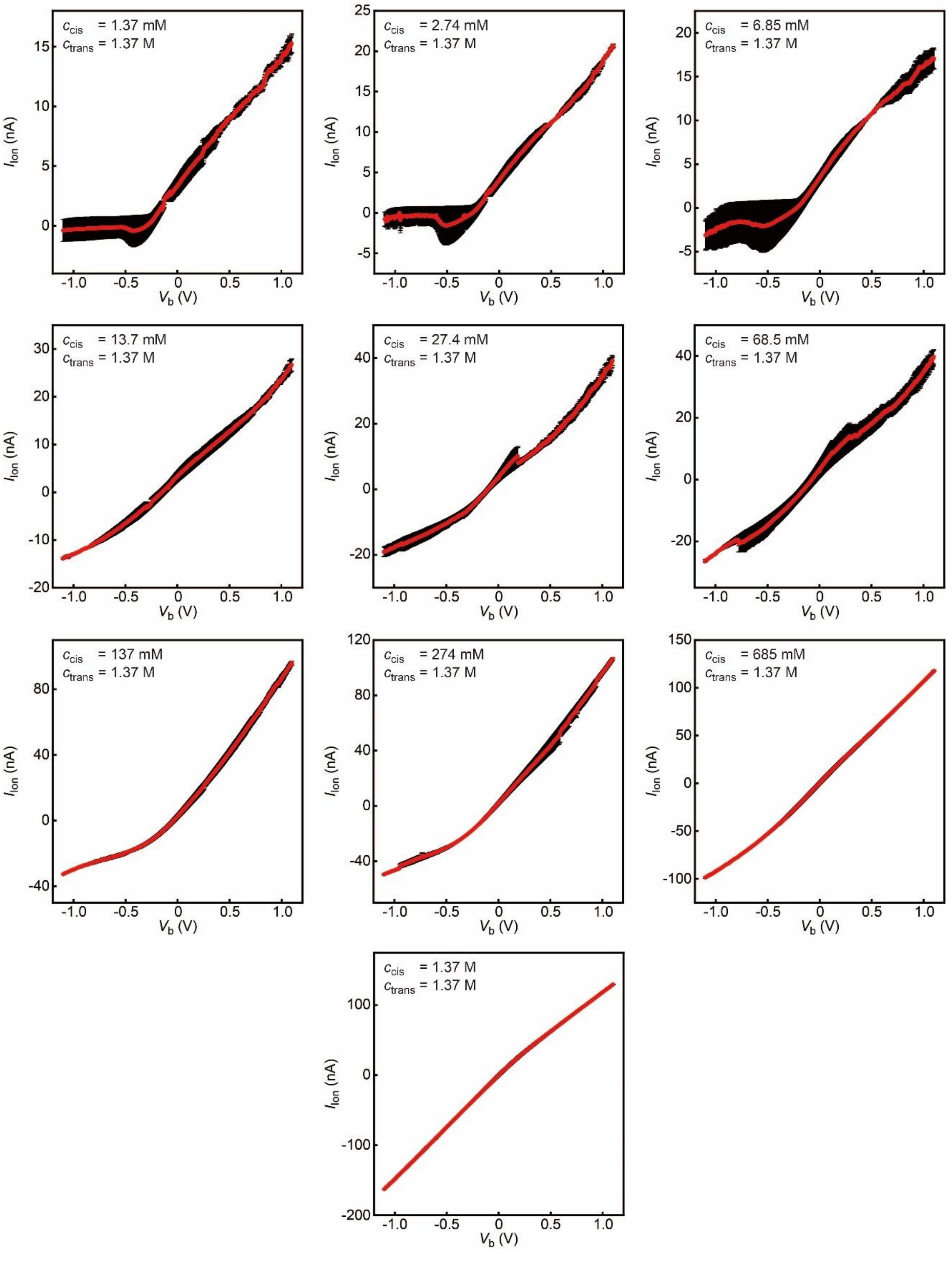

Figure S18. Ionic current (I_{ion}) versus transmembrane voltage (V_b) characteristics of two 20 nm-sized nanopores separated by 30 nm in a 40 nm-thick SiN_x membrane under various salt gradients. The ion concentration at *cis* (c_{cis}) and *trans* (c_{trans}) denote the salinity difference across the membranes. Red plots are the average I_{ion} estimated from the data obtained by scanning V_b from +1 to -1 V and -1 to +1 V. Error bars show the standard deviations.

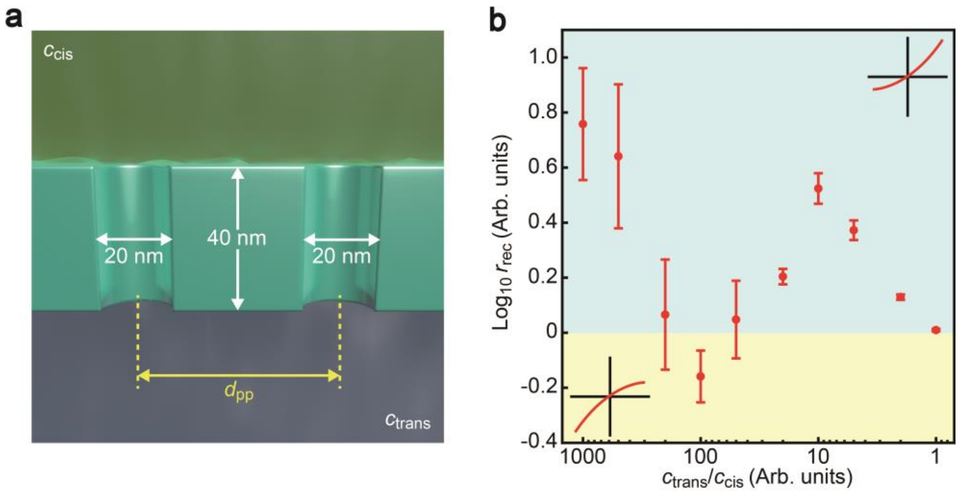

Figure S19. Rectification ratio of the ionic current characteristics of pair pore systems. **a,** A sketch of two cylindrical channels of 20 nm diameter in a 40 nm-thick SiN_x membrane. The distance between the centers of the nanopores d_{pp} is varied from 5000 nm to 30 nm. **b,** r_{rec} as a function of the salt concentration ratio c_{trans}/c_{cis}. Error bars are the standard deviation of the rectification ratio estimated from the ionic current characteristics of the pair nanopores of various d_{pp}.

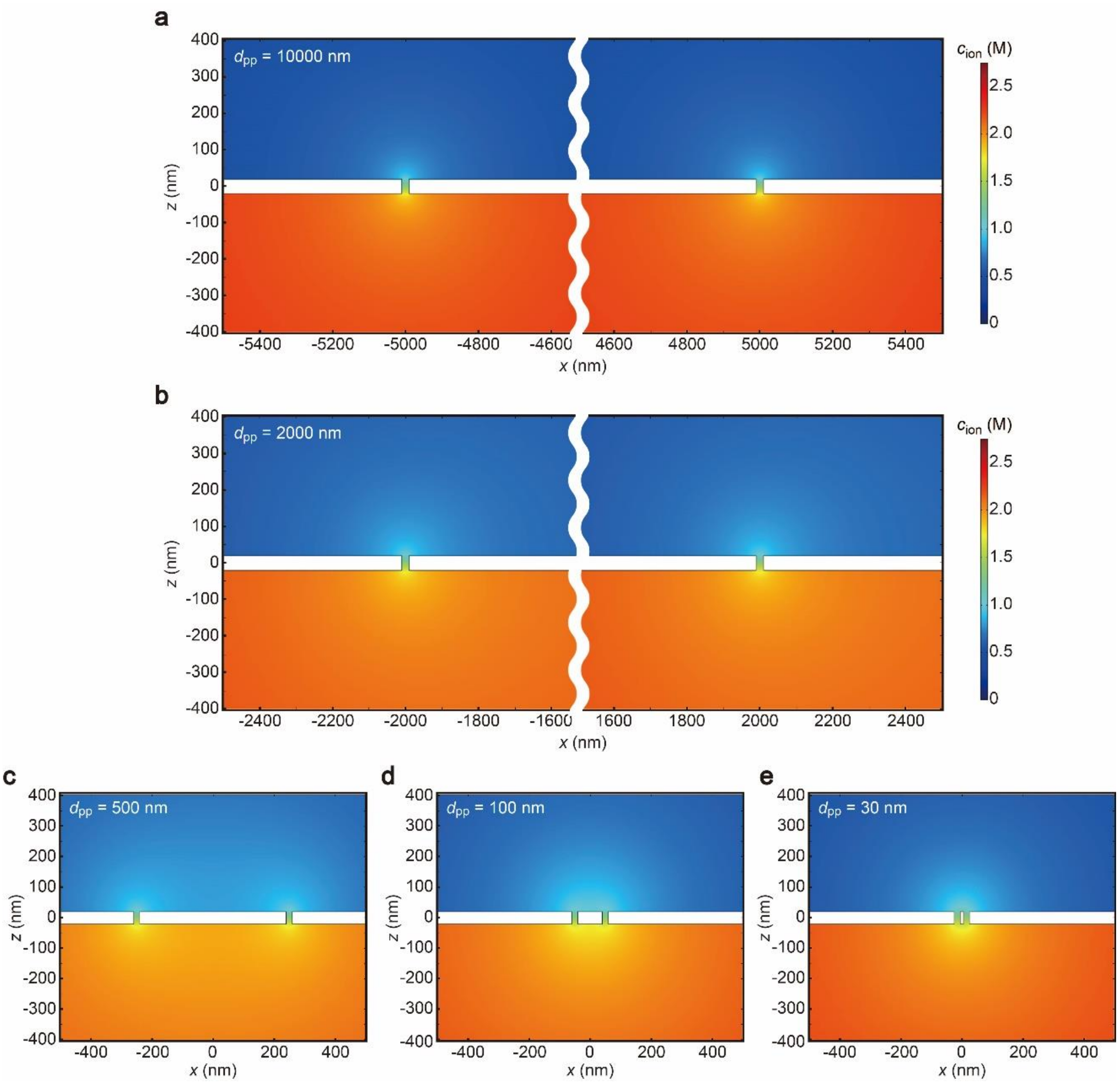

Figure S20. Theoretically-deduced ion concentration distributions around 20 nm-sized pair nanopores in a 40 nm-thick SiN_x membrane under 1000-fold salt concentration difference between *cis* and *trans*. a-e, Heat maps of the ion concentrations of the pair-pore systems with inter-pore distances d_{pp} of 10000 nm (a), 2000 nm (b), 500 nm (c), 100 nm (d), and 30 nm (e).

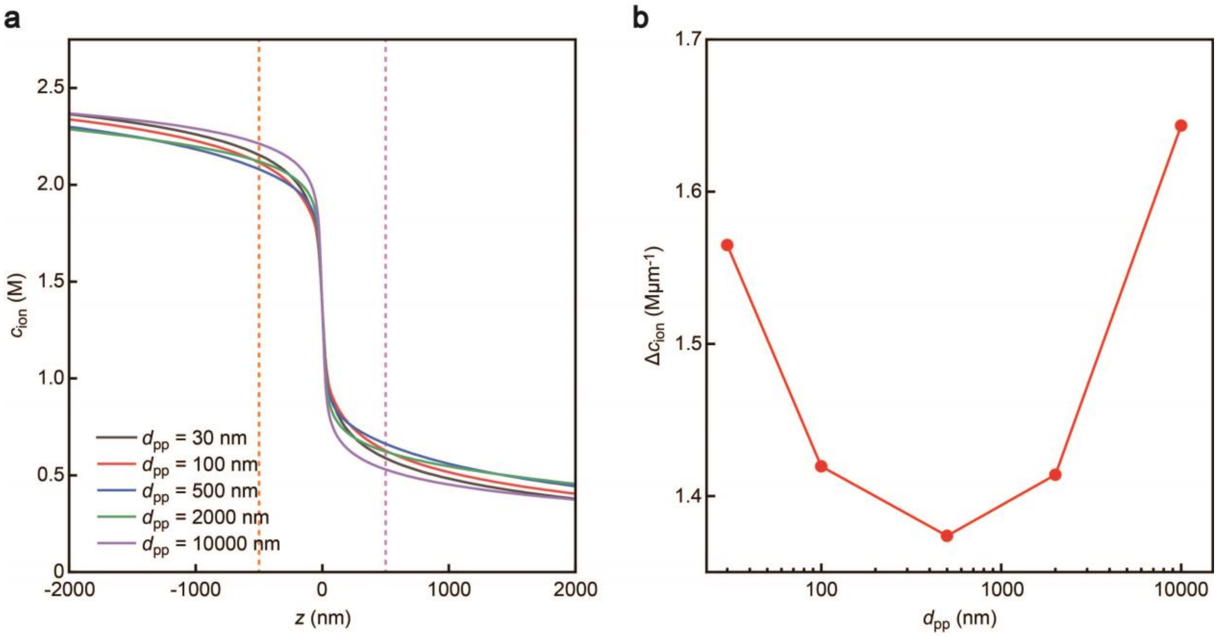

Figure S21. Inter-pore distance-dependent ion concentration gradients. a, The ion concentration c_{ion} along the axial direction of the left-side of the 20 nm nanopores in Figure S17. Orange and purple dashed lines denote z = - 500 nm and +500 nm, respectively. **b,** The concentration gradients Δc_{ion} calculated from c_{ion} at z = +500 nm and -500 nm. Note that Δc_{ion} first decreases but then decreases with reducing inter-channel distance d_{pp}, which is in fair agreement with the non-trivial d_{pp} dependence of the permselectivity observed in the experiments (see Figure 3d in the main text).

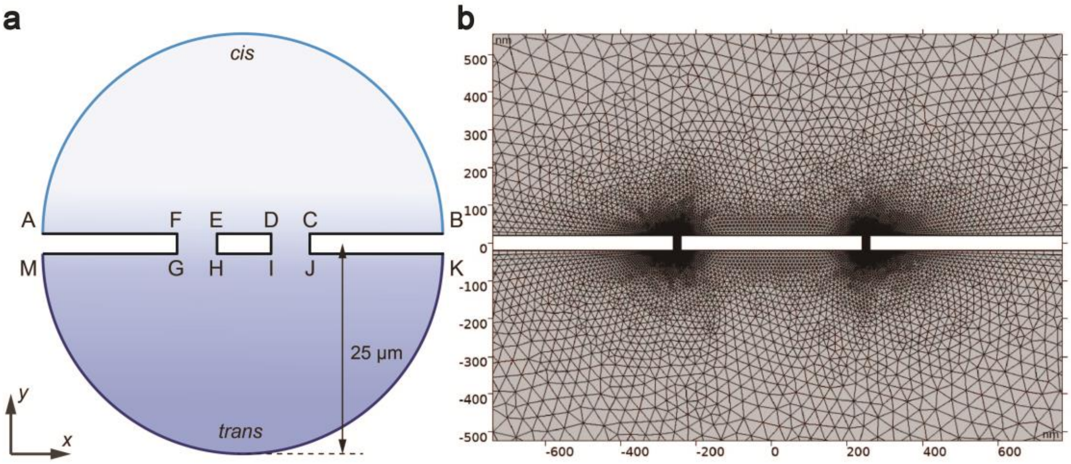

c

Boundaries	AC/ DC	Creeping fluid flow	Transport of diluted species
AB	Grounded ($\Phi = 0$)	Bulk concentration ($c = c_{cis}$)	No slip ($v = 0$)
KM	Applied terminal bias ($\Phi = V_b$)	Bulk concentration ($c = c_{trans}$)	
BC, CJ, JK<br>DE, EH, HJ, DI<br>AF, FG, GM	Constant surface charge density (σ_{mem})	Zeta potential (ζ)	Ion impenetrable ($nN_i = 0$)

Figure S22. Model and conditions used for simulations of the salt concentration distributions around the salinity gradient-applied pair nanopores. a-b, Geometry of the pair-pore system (a, not to scale) and the actual model with meshes (b). **c,** Boundary conditions for the regions A through M defined in (a). Φ, σ, c, n, N_i, μ_{EO}, p, and v are the surface potential, surface charge density of the pore wall and membrane surface, ion concentration, normal vector, ion flux, electroosmotic mobility, pressure, and fluid velocity, respectively. Zeta potential ζ is deduced from σ_{mem} = -15 mCm^{-2} by Graham equation.[S1]

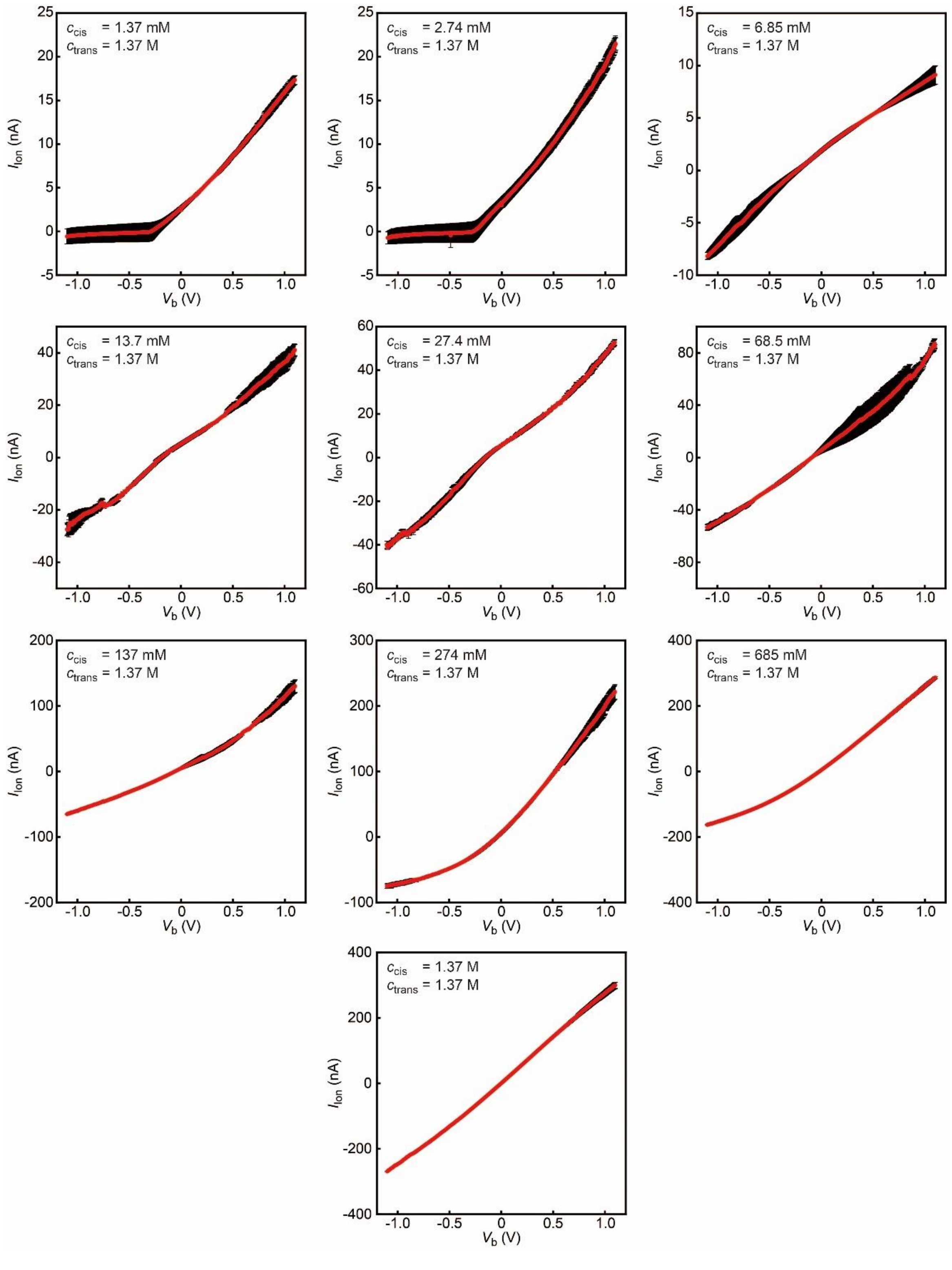

Figure S23. Ionic current (I_{ion}) versus transmembrane voltage (V_b) characteristics of a 2 x 2 array of 20 nm-sized nanopores in a 40 nm-thick SiN_x membrane under various salt gradients. Inter-pore spacing is 9000 nm. The ion concentration at *cis* (c_{cis}) and *trans* (c_{trans}) denote the salinity difference across the membranes. Red plots are the average I_{ion} estimated from the data obtained by scanning V_b from +1 to -1 V and -1 to +1 V. Error bars show the standard deviations.

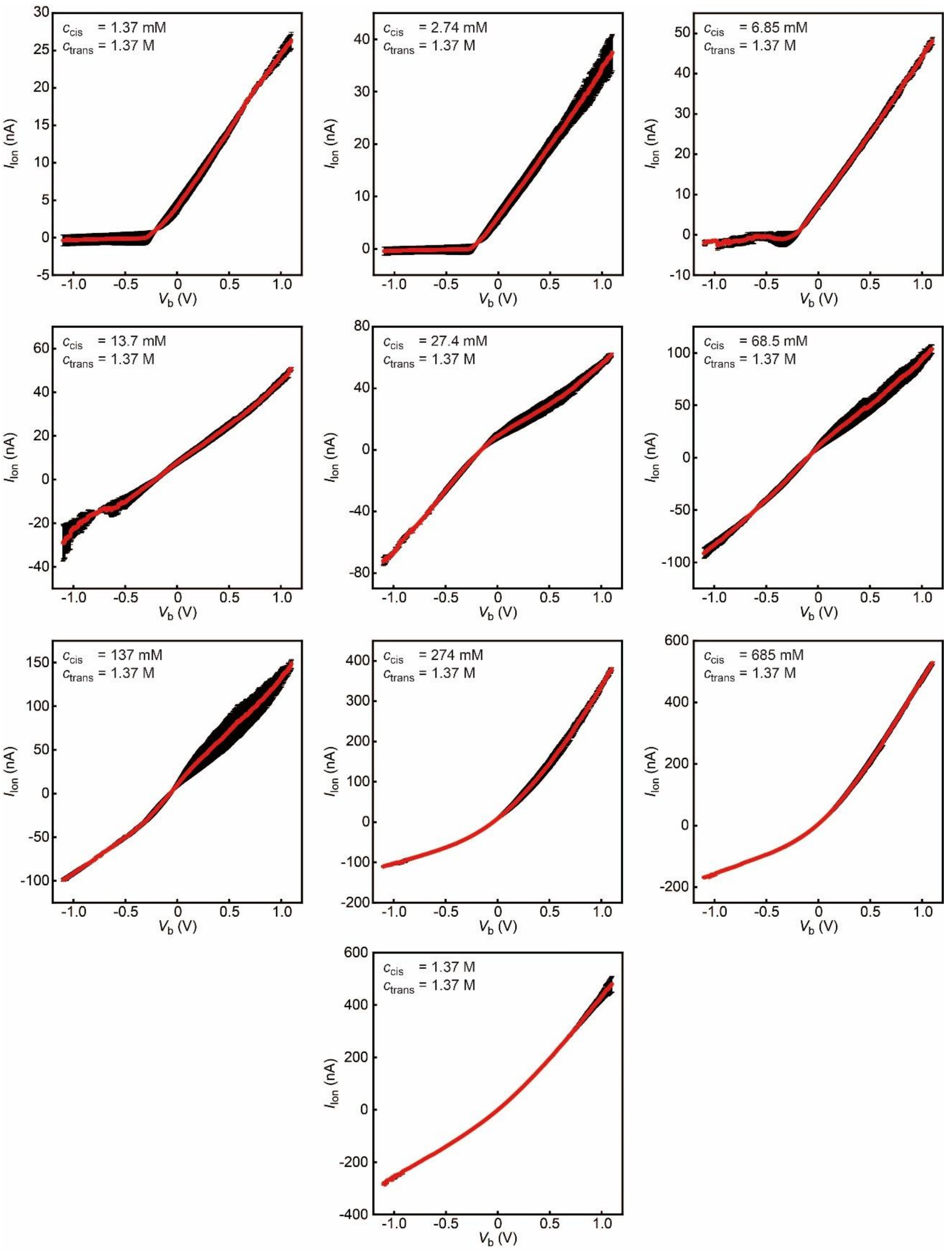

Figure S24. Ionic current (I_{ion}) versus transmembrane voltage (V_b) characteristics of a 4 x 4 array of 20 nm-sized nanopores in a 40 nm-thick SiN_x membrane under various salt gradients. Inter-pore spacing is 3000 nm. The ion concentration at *cis* (c_{cis}) and *trans* (c_{trans}) denote the salinity difference across the membranes. Red plots are the average I_{ion} estimated from the data obtained by scanning V_b from +1 to -1 V and -1 to +1 V. Error bars show the standard deviations.

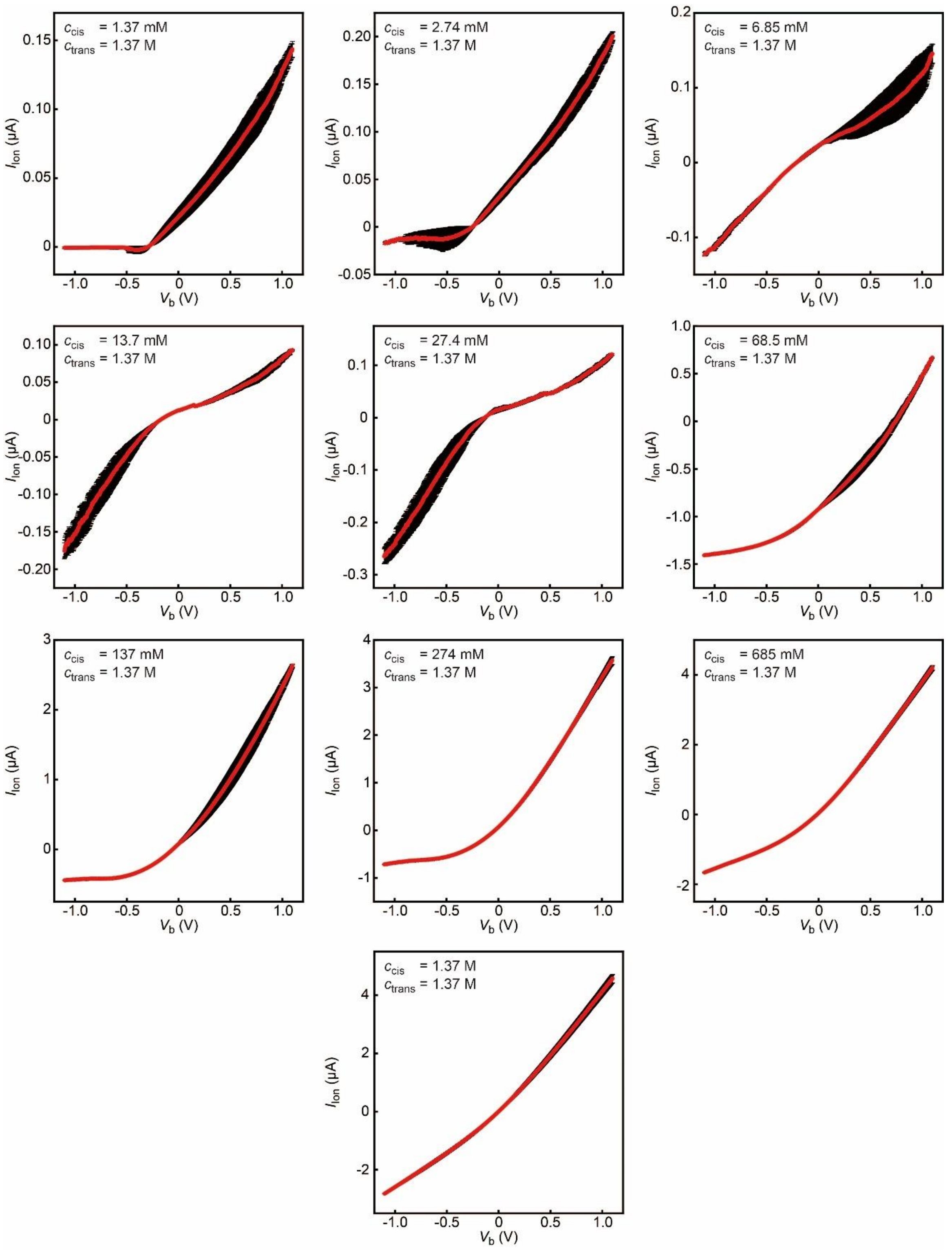

Figure S25. Ionic current (I_{ion}) versus transmembrane voltage (V_b) characteristics of a 8 x 8 array of 20 nm-sized nanopores in a 40 nm-thick SiN_x membrane under various salt gradients. Inter-pore spacing is 1286 nm. The ion concentration at *cis* (c_{cis}) and *trans* (c_{trans}) denote the salinity difference across the membranes. Red plots are the average I_{ion} estimated from the data obtained by scanning V_b from +1 to -1 V and -1 to +1 V. Error bars show the standard deviations.

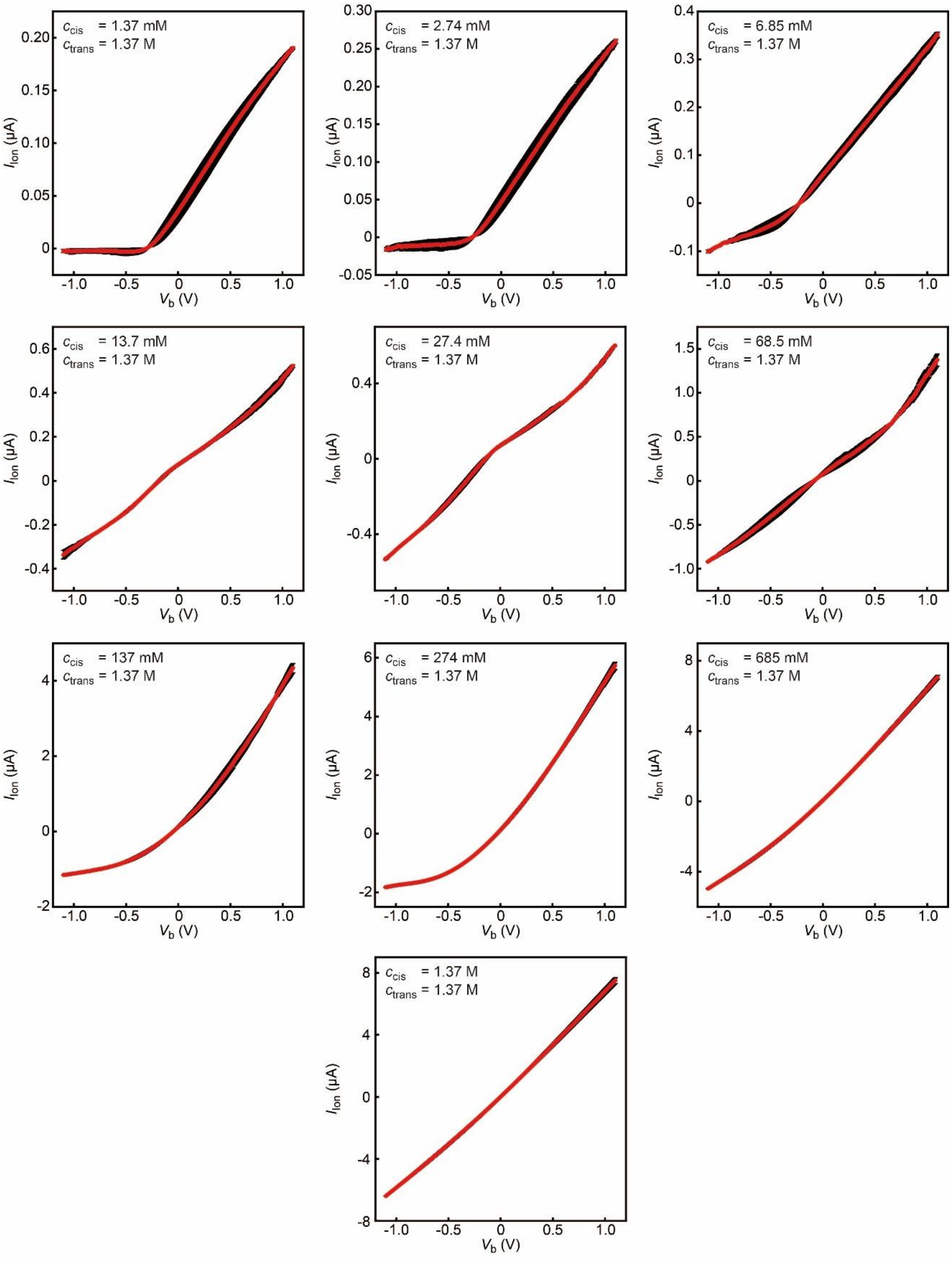

Figure S26. Ionic current (I_{ion}) versus transmembrane voltage (V_b) characteristics of a 10 x 10 array of 20 nm-sized nanopores in a 40 nm-thick SiN_x membrane under various salt gradients. Inter-pore spacing is 1000 nm. The ion concentration at *cis* (c_{cis}) and *trans* (c_{trans}) denote the salinity difference across the membranes. Red plots are the average I_{ion} estimated from the data obtained by scanning V_b from +1 to -1 V and -1 to +1 V. Error bars show the standard deviations.

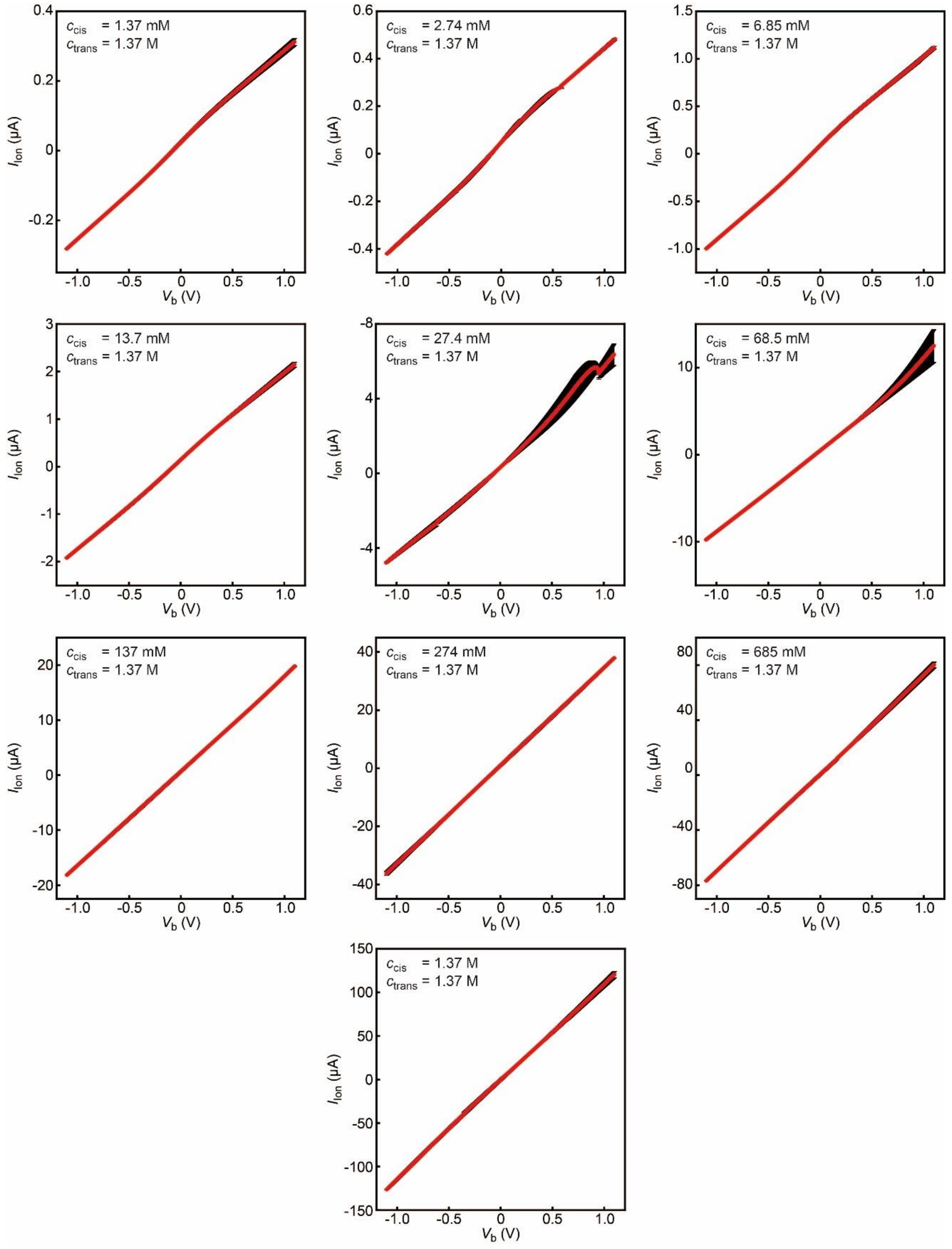

Figure S27. Ionic current (I_{ion}) versus transmembrane voltage (V_b) characteristics of a 20 x 20 array of 20 nm-sized nanopores in a 40 nm-thick SiN_x membrane under various salt gradients. Inter-pore spacing is 474 nm. The ion concentration at *cis* (c_{cis}) and *trans* (c_{trans}) denote the salinity difference across the membranes. Red plots are the average I_{ion} estimated from the data obtained by scanning V_b from +1 to -1 V and -1 to +1 V. Error bars show the standard deviations.

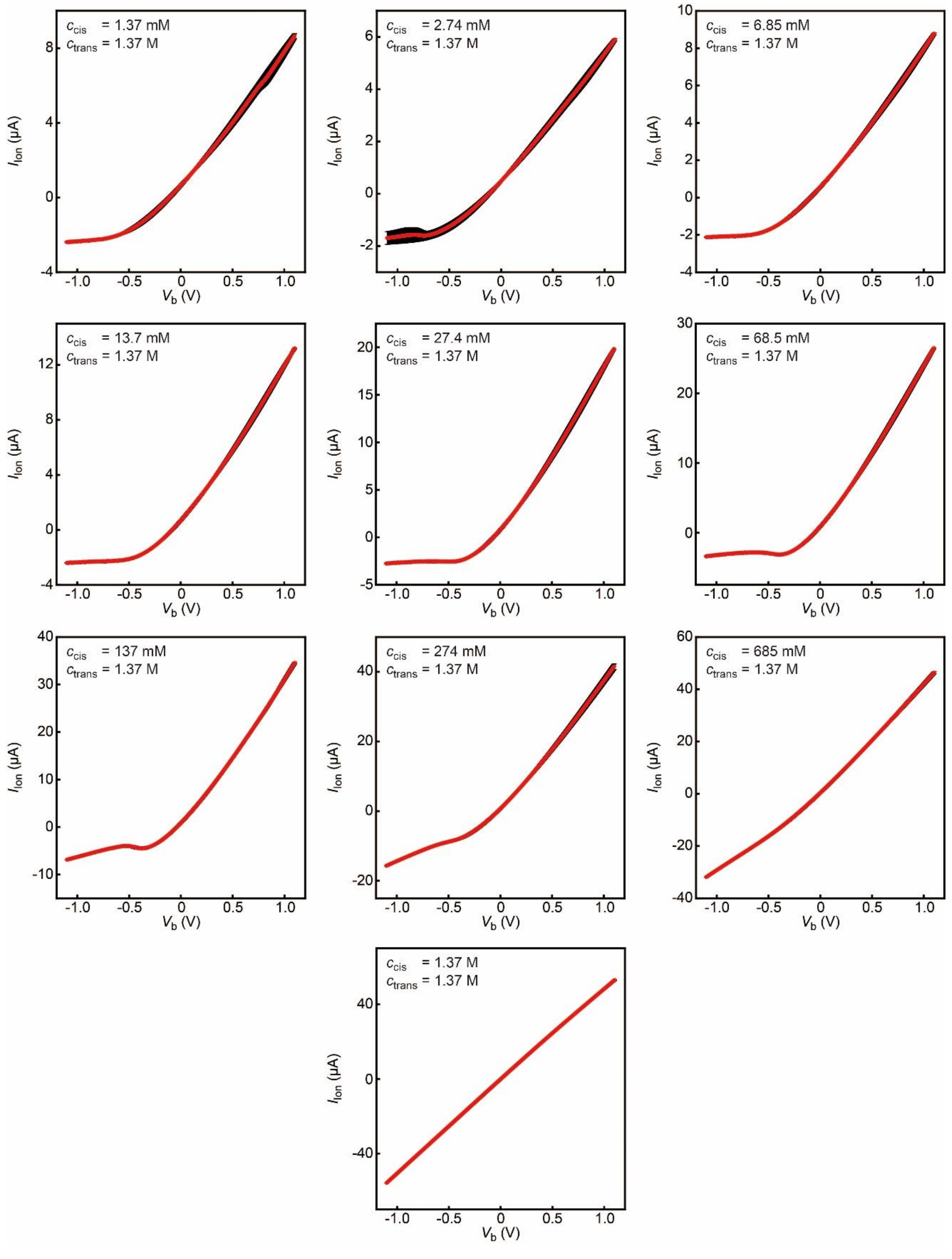

Figure S28. Ionic current (*I*ion) versus transmembrane voltage (*V*b) characteristics of a 40 x 40 array of 20 nm-sized nanopores in a 40 nm-thick SiNx membrane under various salt gradients. Inter-pore spacing is 231 nm. The ion concentration at *cis* (c_{cis}) and *trans* (c_{trans}) denote the salinity difference across the membranes. Red plots are the average I_{ion} estimated from the data obtained by scanning V_b from +1 to -1 V and -1 to +1 V. Error bars show the standard deviations.

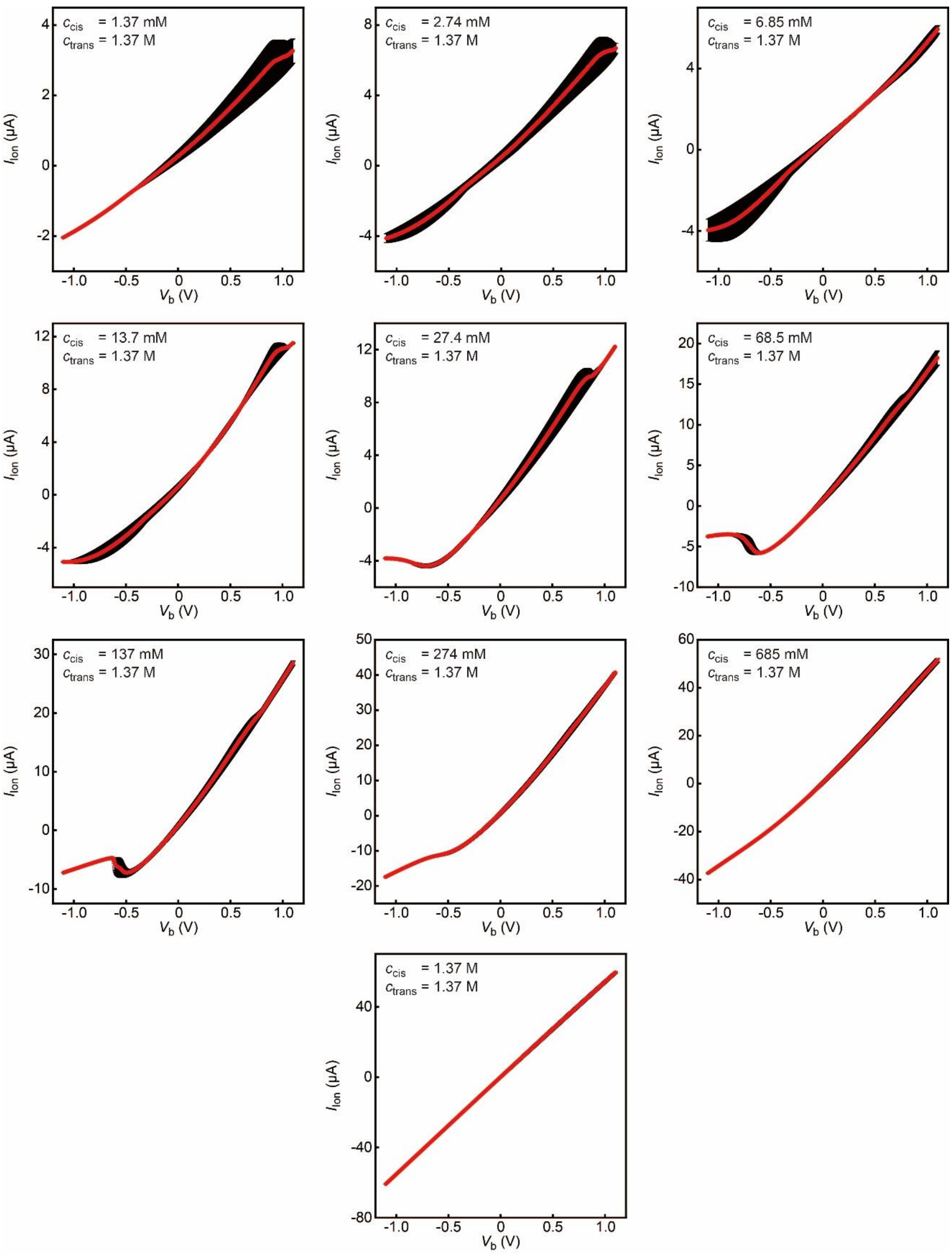

Figure S29. Ionic current (I_{ion}) versus transmembrane voltage (V_b) characteristics of a 60 x 60 array of 20 nm-sized nanopores in a 40 nm-thick SiN_x membrane under various salt gradients. Inter-pore spacing is 153 nm. The ion concentration at *cis* (c_{cis}) and *trans* (c_{trans}) denote the salinity difference across the membranes. Red plots are the average I_{ion} estimated from the data obtained by scanning V_b from +1 to -1 V and -1 to +1 V. Error bars show the standard deviations.

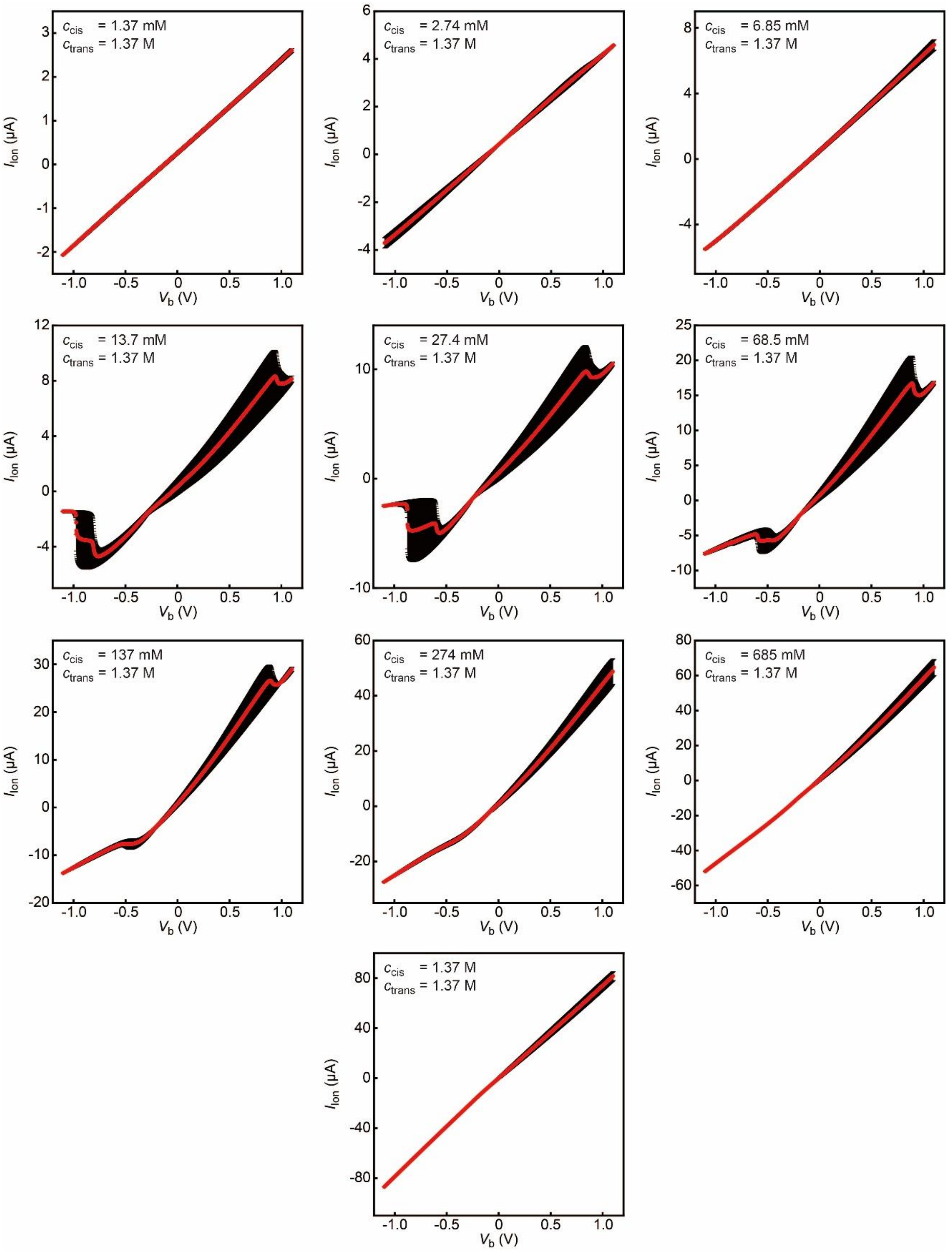

Figure S30. Ionic current (I_{ion}) versus transmembrane voltage (V_b) characteristics of a 80 x 80 array of 20 nm-sized nanopores in a 40 nm-thick SiN_x membrane under various salt gradients. Inter-pore spacing is 114 nm. The ion concentration at *cis* (c_{cis}) and *trans* (c_{trans}) denote the salinity difference across the membranes. Red plots are the average I_{ion} estimated from the data obtained by scanning V_b from +1 to -1 V and -1 to +1 V. Error bars show the standard deviations.

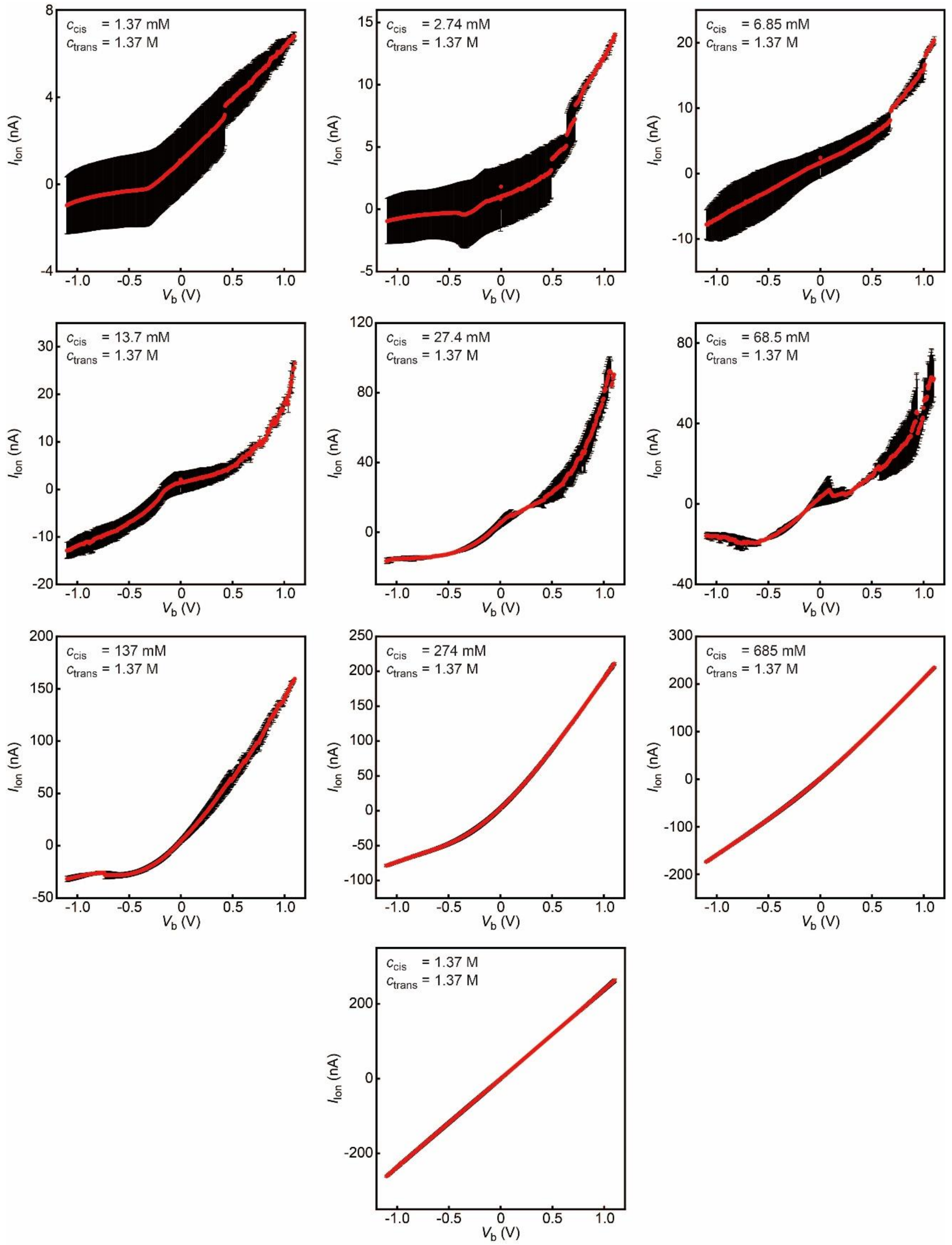

Figure S31. Ionic current (I_{ion}) versus transmembrane voltage (V_b) characteristics of a 100 x 100 array of 20 nm-sized nanopores in a 40 nm-thick SiN_x membrane under various salt gradients. Inter-pore spacing is 91 nm. The ion concentration at *cis* (c_{cis}) and *trans* (c_{trans}) denote the salinity difference across the membranes. Red plots are the average I_{ion} estimated from the data obtained by scanning V_b from +1 to -1 V and -1 to +1 V. Error bars show the standard deviations.

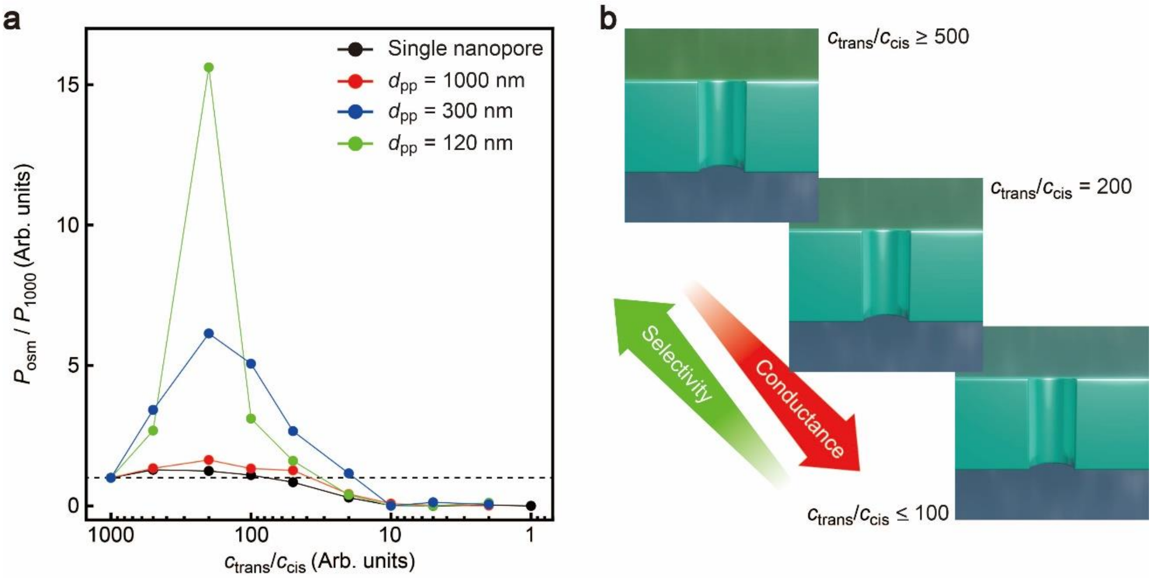

Figure S32. Optimal salinity difference for gaining maximal osmotic power output from pair-pore membranes. The osmotic power P_{osm} at different salinity gradients normalized by that at c_{trans}/c_{cis} = 1000 (P_{1000}). Dotted line points to P_{osm}/P_{1000} = 1. The results show maximum P_{osm} at 200-fold salinity difference irrespective of the inter-pore distance d_{pp}. The trend was also the same for the single-nanopore. **b,** It suggests the fact that the permselectivity remains to be high upon enlarging c_{cis} from 1.37 mM to 6.85 mM, thereby offering the large osmotic power by the gain in the ionic conductance.

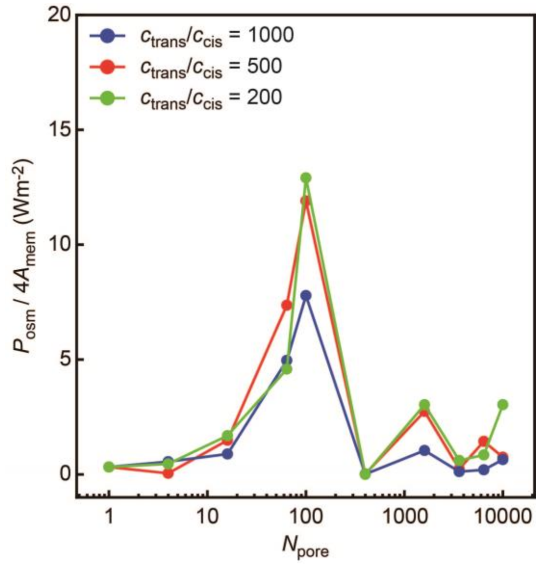

Figure S33. Optimal salinity difference for gaining maximal osmotic power output from multipore membranes. The porosity dependence of the maximum osmotic power density $P_{osm}/4A_{mem}$ under different salinity gradients. The results show maximum power density of 14 Wm^{-2} at 200-fold salinity difference with the number of nanopores N_{pore} = 100.

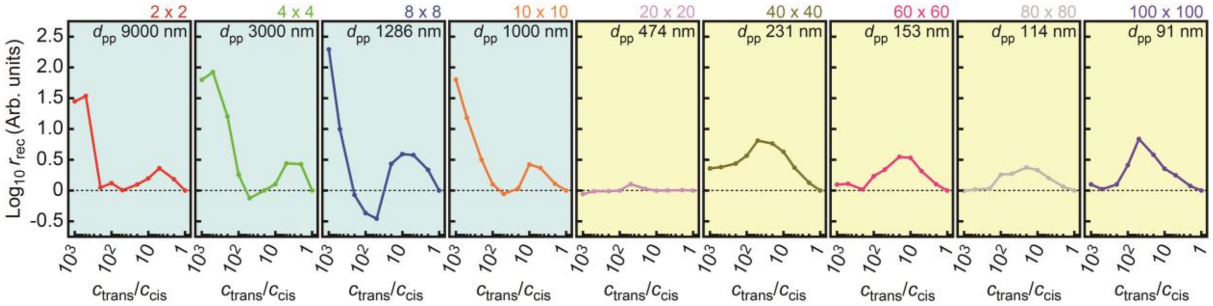

Figure S34. Rectification ratio of the ionic current characteristics of two-dimensional arrays of 20 nm-sized nanopores in 40 nm-thick SiN_x membranes. Change in the ionic rectifying behaviors upon increasing the membrane porosity. Dashed line points to $\log_{10} r_{rec}$ = 0 that denotes no Iion rectification. Blue and yellow colors denote a transition of the behaviors from permselective to non-selective ion transport characteristics upon increasing the number of pores from 100 to 400.

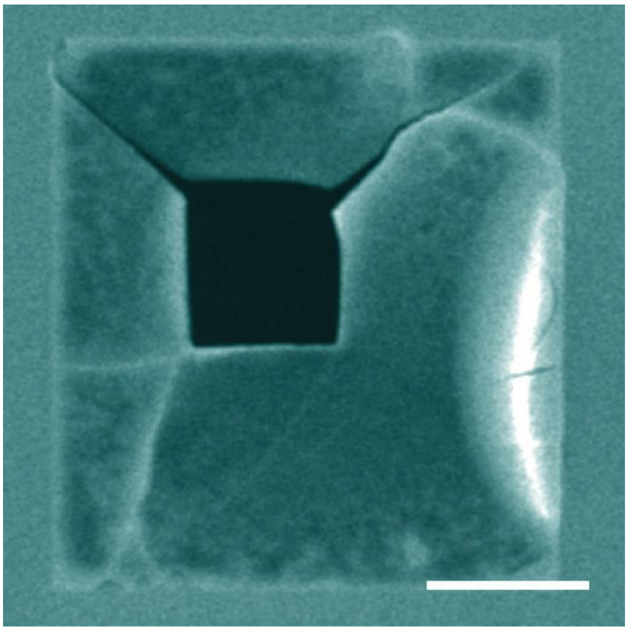

Figure S35. Scanning electron micrograph of a 200x200 two-dimensional array of 20 nm-sized nanopores in a 40 nm-thick SiN_x membrane. The entire multipore region of 9 μm square broke after the reactive ion etching process to sculpt the multipores due presumably to the too-narrow structures between the pores to endure the internal stress in the CVD-formed SiN_x layer (inter-pore distance was about 45 nm). Scale bar denotes 10 μm.

Table S1. Electrode potential difference V_{ele} under salinity difference across membranes obtained from the open circuit voltage of a 10 μm-sized micropore in a 40 nm-thick SiN_x membrane.

c_{trans} (M)	c_{cis} (M)	c_{trans} / c_{cis}	V_{ele} (mV)
1.37	1.37	1	0
1.37	0.69	2	-12.8
1.37	0.27	5	-22.9
1.37	0.14	10	-41.3
1.37	0.069	20	-49.6
1.37	0.027	50	-59.2
1.37	0.014	100	-69.8
1.37	0.0069	200	-81.3
1.37	0.0027	500	-98.7
1.37	0.0014	1000	-128

Supplementary Reference

S1. N. Arjmandi, W. V. Roy, L. Lagae, G. Borghs, Measuring the electric charge and zeta potential of nanometer-sized objects using pyramidal-shaped nanopores. *Anal. Chem.* **2012**, *84*, 8490-8496.
